# Inflammatory Bowel Disease–Associated Changes in the Gut: Focus on Kazan Patients

**DOI:** 10.1093/ibd/izaa188

**Published:** 2020-08-07

**Authors:** Giuseppe Lo Sasso, Lusine Khachatryan, Athanasios Kondylis, James N D Battey, Nicolas Sierro, Natalia A Danilova, Tatiana V Grigoryeva, Maria I Markelova, Dilyara R Khusnutdinova, Alexander V Laikov, Ilnur I Salafutdinov, Yulia D Romanova, Mariia N Siniagina, Ilya Yu Vasiliev, Eugenia A Boulygina, Valeriya V Solovyeva, Ekaterina E Garanina, Kristina V Kitaeva, Konstantin Y Ivanov, Darja S Chulpanova, Konstantin S Kletenkov, Alina R Valeeva, Alfiya Kh Odintsova, Maria D Ardatskaya, Rustam A Abdulkhakov, Nikolai V Ivanov, Manuel C Peitsch, Julia Hoeng, Sayar R Abdulkhakov

**Affiliations:** 1 Philip Morris International Research and Development, Philip Morris Products S.A., Neuchâtel, Switzerland; 2 Kazan Federal University, Institute of Fundamental Medicine and Biology, Kazan, Tatarstan, Russian Federation; 3 Department of Clinical Immunology and Allergology, Kazan State Medical University, Kazan, Tatarstan, Russian Federation; 4 Department of Gastroenterology, Republican Clinical Hospital of Tatarstan Republic, Kazan, Tatarstan, Russian Federation; 5 Central State Medical Academy of Administrative Department of the President of the Russian Federation, Moscow, Russian Federation

**Keywords:** inflammatory bowel disease, microbial dysbiosis, short-chain fatty acids

## Abstract

**Background:**

Several studies have highlighted the role of host–microbiome interactions in the pathogenesis of inflammatory bowel disease (IBD), resulting in an increasing amount of data mainly focusing on Western patients. Because of the increasing prevalence of IBD in newly industrialized countries such as those in Asia, the Middle East, and South America, there is mounting interest in elucidating the gut microbiota of these populations. We present a comprehensive analysis of several IBD-related biomarkers and gut microbiota profiles and functions of a unique population of patients with IBD and healthy patients from Kazan (Republic of Tatarstan, Russia).

**Methods:**

Blood and fecal IBD biomarkers, serum cytokines, and fecal short-chain fatty acid (SCFA) content were profiled. Finally, fecal microbiota composition was analyzed by 16S and whole-genome shotgun sequencing.

**Results:**

Fecal microbiota whole-genome sequencing confirmed the presence of classic IBD dysbiotic features at the phylum level, with increased abundance of Proteobacteria, Actinobacteria, and Fusobacteria and decreased abundance of Firmicutes, Bacteroidetes, and Verrucomicrobia. At the genus level, the abundance of both fermentative (SCFA-producing and hydrogen (H_2_)-releasing) and hydrogenotrophic (H_2_-consuming) microbes was affected in patients with IBD. This imbalance was confirmed by the decreased abundance of SCFA species in the feces of patients with IBD and the change in anaerobic index, which mirrors the redox status of the intestine.

**Conclusions:**

Our analyses highlighted how IBD-related dysbiotic microbiota—which are generally mainly linked to SCFA imbalance—may affect other important metabolic pathways, such as H_2_ metabolism, that are critical for host physiology and disease development.

## INTRODUCTION

Inflammatory bowel diseases (IBD) are a group of disorders characterized by chronic inflammation of the gastrointestinal tract. They present with 2 main manifestations, ulcerative colitis (UC) and Crohn disease (CD), each with distinctive clinical and pathological features but both exerting a substantial impact on the quality of life of patients and on medical costs. The prevalence of IBD in Western countries is estimated to be 0.5% in the general population, with a steady increase expected over the next decade. Several studies have established that IBD prevalence in newly industrialized countries parallels the patterns observed in the Western world during the early 20th century. This new trend coincided with the beginning of globalization and the “Westernization” of Asian, South American, Eastern, and Middle Eastern societies, highlighting the fundamental role of environmental factors in accelerating the onset of IBD.^1^ The etiology of IBD is not fully understood, and the term is used to describe a collection of chronic immune-mediated diseases of unknown, multifactorial etiology with complex interactions between genetic and environmental factors. One commonality among the diseases is an abnormal immune response to antigens of commensal microorganisms that is modulated by environmental and clinical factors such as smoking, diet, drugs, geographical area, stress, microbial agents, intestinal permeability, and appendectomy.^2^

The role of host–microbe interactions in the pathogenesis of IBD has been highlighted by genetic studies. One of the most recent and largest genetic association studies, which employed genome-wide association data for >75,000 patients with IBD and control patients, identified 163 susceptibility loci for IBD, including approximately 300 potential candidate genes.^3^ Of these loci, 110 conferred risk to both IBD subtypes, 30 were unique to CD, and 23 were exclusive to UC. Interestingly, mutation of the associated genes often affects specific host pathways linked to microbial response in IBD, such as the *NOD2*-mediated innate immune response to bacterial infections, the interleukin (IL)-23 pathway, autophagy, and Paneth cell functions.^4^

Over the past decade, technological advances in high-throughput DNA sequencing methods have enhanced the characterization of the gastrointestinal microbiome in both healthy patients and those with IBD. The human gut harbors more than 1000 bacterial species, with more than 99% belonging to 4 phyla: Firmicutes, Bacteroidetes, Proteobacteria, and Actinobacteria.^5^ Data from experimental models and clinical studies^6^ and the recent outcomes reported by the Integrative Human Microbiome Project^7^ have shown a clear imbalance or dysbiosis in the microbiota of patients with IBD, with a global decrease in biodiversity, a decrease in the proportions of Firmicutes and Bacteroidetes relative to the proportions of Proteobacteria and Actinobacteria. The changes in the composition of gut microbiota in IBD studied at the genus or species taxonomic levels have displayed specific features; thus, although the abundance of genera such as *Faecalibacterium, Roseburia, Ruminococcus,* and *Akkermansia* is generally lower in IBD, *Escherichia, Enterococcus,* and *Streptococcus* abundance is higher.^8^

However, evidence suggests that the complex host–microbiome interaction can be understood better by using a holistic approach. Thus, focusing on the functions and metabolic activities of a microbe or dysbiotic community can bridge the gap between gut microbiota and IBD pathogenesis. One example of the importance of the influence of microbiome metabolic activity on host health is the digestion of dietary fiber, which is used by microbiota as a source of energy. Carbohydrates resulting from the degradation of polysaccharides in fiber are fermented into short-chain fatty acids (SCFAs) such as acetate, propionate, and butyrate. The SCFAs are a fundamental source of energy for intestinal epithelial cells, which have a gut barrier function and important immunomodulatory functions. Bacteria that ferment fiber and produce SCFAs are known to be significantly reduced in abundance in the mucosa and feces of patients with IBD.^9^

The intestinal microbiota of IBD populations from different regions such as Europe, the United States, and China have been studied extensively, resulting in large metagenomics datasets.^10, 11^ Yet metagenomics studies in the Russian population are either underrepresented or relevant only to healthy cohorts.^12, 13^ In the present study, we profiled and compared blood and fecal IBD biomarkers, fecal SCFAs, fecal microbial communities, and their functional roles in patients with IBD and healthy control patients from Kazan (Republic of Tatarstan, Russia). The microbiota composition in this population may be of particular interest: With more than 150 ethnicities, the Republic of Tartastan represents a unique example of a multiethnic and multilifestyle Eastern European country. Our study revealed differences in biomarkers and microbiome composition among patients with UC, CD, and healthy control patients and may contribute to the identification of novel molecular and microbiome-based signatures and approaches for the clinical management of IBD.

## METHODS

### Patients

A total of 126 patients (42 healthy volunteers, 41 patients with CD, and 43 patients with UC) who regularly visited the Republican Clinical Hospital (Kazan, Russian Federation) and the Kazan Federal University Hospital (Kazan, Russian Federation) between 2014 and 2018 were recruited to the study. Patients with IBD were diagnosed on the basis of standard clinical, endoscopic, and histological criteria. Endoscopic data are currently not available per se: During the COVID-19 outbreak in the Russian Federation, the Republican Clinical Hospital and the Kazan Federal University Hospital have been converted into COVID-19 hospitals, thus making it temporarily impossible to recover such data from the archives. The eligibility of patients with IBD and healthy volunteers was determined in accordance with specific inclusion/exclusion criteria, as reported in Supplementary Table 1. Written informed consent was obtained from all patients before study enrollment. To reduce the impact of diet heterogeneity in our study, all patients with IBD were asked to follow specific diet indications^14^ starting from the first visit to the gastroenterologist throughout the course of disease. The study was reviewed and approved by the local ethics committee of the Kazan Federal University, Kazan, Russia.

### Molecular Markers and SCFAs

#### Detection of stool biomarkers

Stool biomarkers were detected using standard ELISA protocols, with the following kits: SEB080Hu for S100A12 (Cloud-Clone), SEA780Hu for lactoferrin (Cloud-Clone), E01N0603 for neopterin (Shanghai BlueGene Biotech Co., Ltd.), and CALPR0170 for calprotectin (Calpro AS).

#### SCFA analysis

The SCFA quantification was performed by gas-liquid chromatographic analysis of fecal samples. Commercially available acetic, propionic, butyric, isobutyric, valeric, isovaleric, caproic, and isocaproic acids were used as standards. The chromatography system was set up with a flame ionization detector and a 30 m quartz capillary. Fecal samples were prepared in sterile tubes, weighed (approximately 2 g per sample), and homogenized in 3 mL of distilled water and 2 mL of α,α-dimethyl oil acid (internal standard). One part of the resulting homogenate was used for gas-liquid chromatography after centrifugation (7000 rpm for 10 min). For analysis of total acids, the remaining part of the homogenate was mixed with 2 mL of 1 N hydrochloric acid, stirred, and centrifuged (7000 rpm for 10 min). Next, aliquots of the 1-µL supersedimentary liquid were injected into the chromatograph evaporator using a microsyringe. Individual acids were quantified (as mg per g of sample) from the resulting chromatograms by using the formula Pn = P’ Sn Kn / S′ Po, where P’ was the weight of the internal standard in the analyzed sample, Sn wasa the peak area of the analyzed acids, S′ was the peak area of the internal standard, Po was the weight of the sample, and Kn was the weighting correction factors. Finally, K2 was acetic acid (2.54 ± 0.01), K3 was propionic acid (1.55 ± 0.01), K4 was butyric and isobutyric acids (1.19 ± 0.01), and K5 was valeric and isovaleric acids (1.08 ± 0.01).

### Metagenomics Analyses

#### DNA extraction, amplification, and sequencing

For both shotgun and 16S rRNA gene sequencing, whole genomic DNA was extracted using the QIAamp DNA Stool Mini Kit (Qiagen) in accordance with the manufacturer’s instructions. The procedure was performed as described in previous research.^15^ Samples were sequenced using an Illumina sequencer at the Interdisciplinary Center of Shared Use of Kazan Federal University (Kazan, Russia; https://www.kpfu.ru).

#### Metagenomic whole-genome sequencing data processing

##### Quality control.

Raw whole-genome sequencing (WGS) reads were mapped on the human genome (hg38) by using the Minimap2 (version 2.8) aligner with the option –a (CIGAR and output alignment in SAM format) and –x sr (short single-end reads without splicing). Unmapped reads were collected by using the SAMtools (version 1.7) *view* command with the –f 12 SAM flag (read unmapped, mate unmapped). The reads thus obtained were subjected to contaminant and adapter trimming using the BBDuc program of the BBTools toolkit (version 37.99) with the *k* size set to 23. Quality control reports for the raw, pre-, and post-trimming reads were generated using the FastQC (version 0.11.6) software and gathered using the MultiQC (version 1.7) module for Python.

##### Taxonomic profiling.

Taxonomic profiling was performed using the Kraken2 software for initial classification and the Bracken tool for subsequent correction of the initial results. The Kraken2 reference database was constructed by using the *—standard* option, which enforces the download of RefSeq bacterial/archaeal genomes, RefSeq plasmid sequences, RefSeq complete viral genomes, and the GRCh38 human genome. The Bracken database was constructed by using 75 as the read length and 35 as the *k*-mer length. Bracken correction was performed with a minimum of 10 reads required for classification at the species, genus, family, and phylum ranks. Within-sample diversity (alpha diversity) was estimated with the Shannon index (H’) by using the following formula:


$$\begin{array}{l} H^{{}^{\prime }}=\underset{i=1}{\overset{R}{\sum }}\;p_{i}*\ln p_{i} \end{array}$$

where *p*_*i*_ represents the relative abundance of the i-th taxa.

#### Statistical and mathematical methods for 16S and WGS metagenomics data analysis

The distribution of taxon abundances in the 2 study groups was compared by the nonparametric Mann-Whitney *U* test. The significance threshold was set to 5% after adjusting for the false discovery rate in multiple testing. The calculations were performed with custom scripts in Python by using the scipy.stats.mannwhitneyu and statsmodels.stats.multitest.multipletests packages. The principal coordinate analysis of the weighted UniFrac distance matrix was performed by using the skbio.stats.ordination.pcoa package for Python, with the number of dimensions set to 3.

Datasets, detailed protocols, and additional data visualizations are available on the INTERVALS platform at https://doi.org/10.26126/intervals.hvqhqm.1.

The below information is found in the Supplementary Material section:

- Enzyme-linked immunosorbent assay;
- Cytokine profile in serum samples;
- Statistical analysis of molecular markers, cytokines, and SCFAs;
- 16S sequencing data analysis; and
- Functional profiling (pathway abundance).

## RESULTS

### Study Design and Population

**FIGURE 1.**
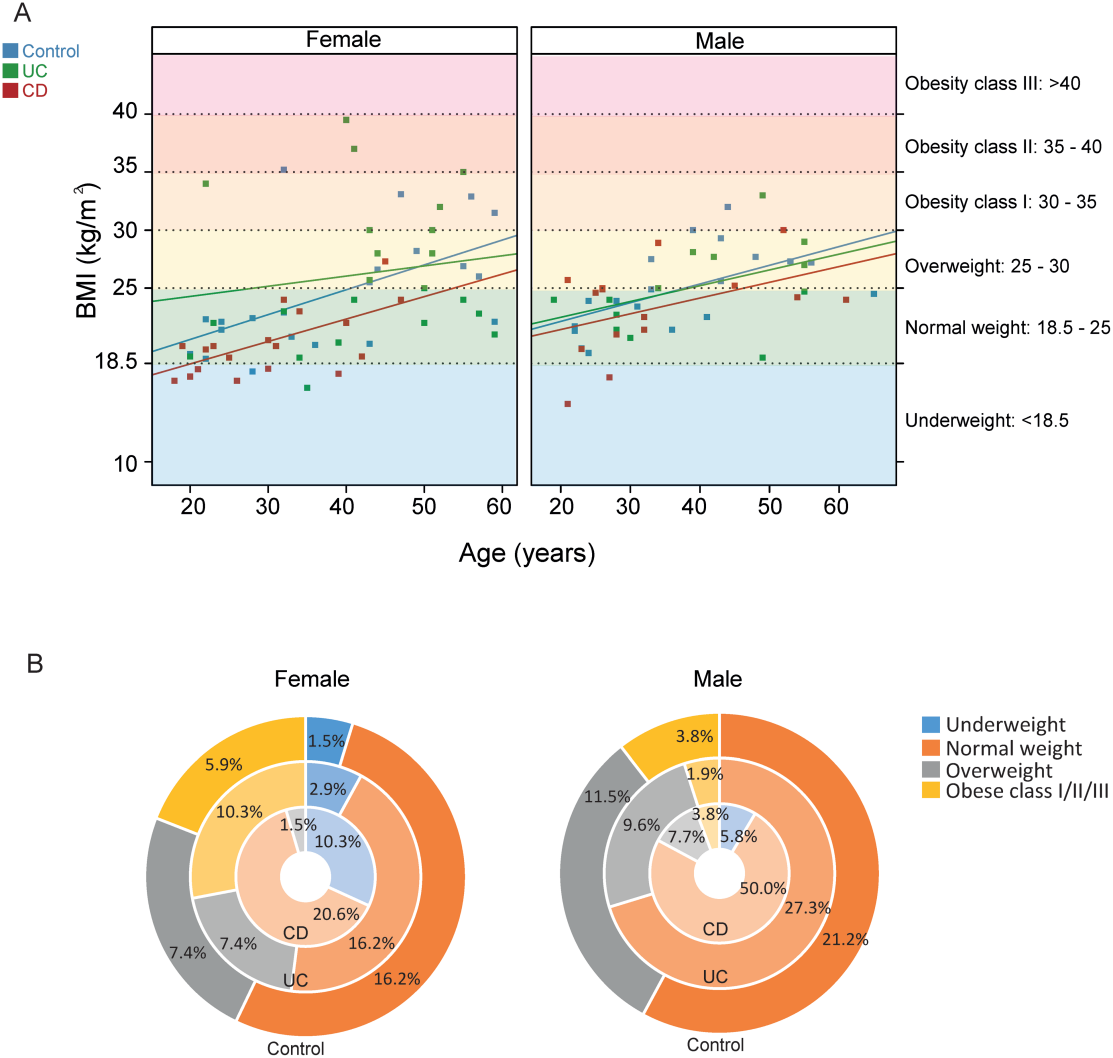
BMI vs age. A, BMI vs age for all patients, colored by study arm. Results depicted separately for female (left panel) and male (right panel) patients. Linear regression lines (illustrated by study arm) relate the evolution of BMI according to age, sex, and study arm. The *y* axis labels use BMI category limits as defined by the WHO for defining nutritional status. B, Pie charts showing the percentages of women (left panel) and men (right panel) in each BMI category (as defined by the WHO) in each study arm (from outer to inner circles, respectively: control patients, patients with UC, and patients with CD). BMI indicates body mass index; WHO, World Health Organization.

To analyze the composition and function of the gut microbiota in healthy patients and patients with IBD, 126 fecal samples were collected from a cross-section of 42 healthy volunteers, 41 patients with CD, and 43 patients with UC, all of whom were nonsmokers. After a quality check, 15 samples were excluded, and the total population analyzed is shown in Table 1 and Supplementary Table 2. In addition to general information such as sex, age, and body mass index, we also collected data on disease activity, treatment, and location of the inflammation. Patients with CD, particularly female patients, had a lower body mass index than patients with UC and the control patients (Fig. 1A). This detail was also confirmed by the percentages of patients categorized by body weight (Fig. 1B and Supplementary Table 3). Thus, the percentage of patients who were underweight was higher in the UC and CD patient groups than in the healthy control patient group. The opposite trend was observed for the percentage of patients who were overweight (Fig. 1B and Supplementary Table 3). Supplementary Fig. 1A shows that this trend was not correlated with disease severity, although these findings may be underestimated because of the low number of patients with moderate CD (n = 6).

**TABLE 1.** Patient Metadata

Groups	Healthy Control Patients	CD	UC
Sample size	40	35	36
Age (y)	38 ± 2	32 ± 2	41 ± 2
Sex			
Male	19	15	14
Female	21	20	22
BMI	24.74 ± 0.69	21.63 ± 0.61	25.83 ± 0.91
Disease activity*			
Inactive (remission)	—	—	1^†^
Mild	—	29	10 (+4)^†^
Moderate	—	6	17 (+4)^†^
Severe	—	—	—
Colitis			
Pancolitis	—	—	16
Left colitis	—	—	12
Proctitis	—	—	8
Ileitis	—	4	
Colitis	—	17	
Ileocolic	—	12	
Valva ileocaecalis	—	2	

*Disease activity calculated according to the Mayo score for UC (0-2, remission/minimal disease activity; 3-5, mild UC; 6-10, moderate UC; and 11-12, severe UC) and the Crohn’s Disease Activity Index for CD (<150, inactive phase; 150-300, mild; 301-450, moderate; >450, severe).

^†^(+n) Patients assigned according to the partial Mayo score. BMI indicates body mass index and is shown as mean ± standard error.

For metagenomics analysis, DNA was extracted from all samples and analyzed by both 16S and whole-genome shotgun sequencing. After a quality check, 2 more samples were excluded. Finally, blood cytokines and fecal IBD biomarkers and SCFAs were measured.

### Markers of Inflammation in the Study Groups

**FIGURE 2.**
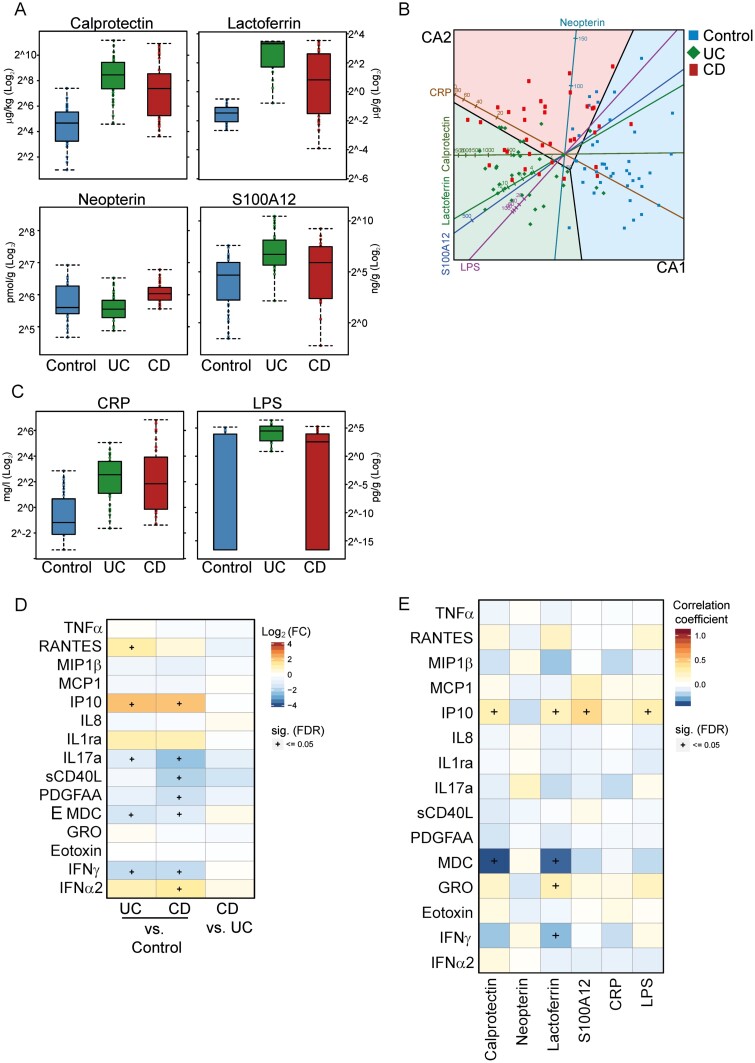
Fecal and serum biomarkers of inflammation. A, Boxplot representing fecal biomarker abundance in the 3 study groups, plotted on a log_2_ scale. From left to right: control patients, patients with UC, and patients with CD. Dots superposed on boxplots to better depict data distribution. Sample medians depicted by arm, using black lines inside the boxes. Lower and upper box limits correspond to first and third quartiles of sample values. B, Canonical variate analysis biplots. Patients (points) and biomarkers of inflammation (axes) simultaneously projected on 2-dimensional canonical axes (CA1 and CA2). Thick solid lines colored by group depict the 0.95-bags for each group, reflecting the deepest 95% core of data for a given group. Prediction regions for each study arm also depicted using study arm colors. C, Boxplot representing abundance of serum biomarkers of inflammation in the 3 study groups, plotted on a log_2_ scale. Sample medians depicted by arm using black lines inside the boxes. Lower and upper box limits correspond to first and third quartiles of sample values. D, Heatmap representing differential cytokine abundance in patients with UC and CD relative to healthy control patients or to each other. E, Correlation between cytokines and biomarkers of inflammation as estimated via correlation coefficients. In heatmaps (D–E), differential abundance or correlation coefficients displayed using an intensity-dependent color map and complemented by their statistical significance (+ = adjusted [false discovery rate] *P* value ≤0.05). Color codes and symbols: blue, control patients; red, patients with CD; green, patients with UC.

Patients with IBD were diagnosed on the basis of the findings of a combination of clinical evaluation, endoscopy, histological examination, and noninvasive analysis of inflammation markers. Fig. 2 and Supplementary Tables 4 and 5 display and list the fecal and serum biomarkers analyzed in this study.

Fecal biomarkers (Fig. 2A and Supplementary Fig. 1B) were higher in patients with UC and those with CD than in healthy control patients. Canonical variate analysis, which helped extract the marker profiles (reflected by the canonical variate components) that best separated the study groups (Fig. 2B), showed a clear separation between patients with UC and those with CD on the first canonical axis owing to the higher levels of neopterin in patients with CD. The increased levels of serum C-reactive protein and lipopolysaccharide concentration in patients with IBD (Fig. 2C and Supplementary Fig. 1B) highlighted the presence and intensity of systemic inflammation and endotoxemia. As previously reported,^16, 17^ the concentration of most of these biomarkers correlated with disease activity in both UC and CD (Supplementary Figs. 2A, B). In particular, calprotectin, lactoferrin, S100A12, and C-reactive protein levels showed a clear difference between mild and moderate disease, with the latter showing higher values.

In Supplementary Table 2, patients with IBD are stratified according to therapeutic intervention. This further stratification showed a high heterogeneity, with the bulk of patients treated using 5-aminosalicylates (14 patients with UC and 8 patients with CD) and the others distributed among other single treatments, such as steroids, immunosuppressors, and biologics, or multi-cotreatments. To understand how the level of inflammatory biomarkers was associated with the therapeutic interventions, patients were divided into 3 groups (Supplementary Table 2): the single-treatment group, in which all patients were receiving only 1 drug (18 patients with UC and 17 patients with CD); the cotreatment group, in which patients were treated using a combination of 2 or 3 drugs (14 patients with UC and 10 patients with CD), and the no-treatment group (2 patients with UC and 2 patients with CD).

Biomarkers of inflammation were represented according to this separation (Supplementary Figs. 3A, B). As shown in Supplementary Figs. 3A and B, for both UC and CD the biomarker values tended toward an increase when cotreatments were present, whereas the patients with no treatment showed values closer to those of the control patients. Furthermore, the association between treatment and disease activity is shown in Supplementary Fig. 3C. Whereas a positive association was observed between the single-treatment group and patients with inactive or mild disease, cotreatment of patients using more than 1 drug was positively associated with moderate disease activity (Supplementary Fig. 3C). Finally, very few patients with IBD were positive for antibodies against *Saccharomyces cerevisiae* (ASCA) or perinuclear anti-neutrophil cytoplasmic antibodies, confirming the low specificity and sensitivity of these assays (Supplementary Tables 4 and 5).

Circulating cytokines were measured. Of the 42 cytokines measured in serum, only 15 were detectable in a consistent number of patients (Fig. 2D). Cytokines with concentrations significantly higher in patients with IBD than in healthy control patients included Regulated upon Activation, Normal T Cell Expressed and Presumably Secreted, interferon gamma-induced protein (IP10), and interferon (IFN)α2, and those with nonsignificantly higher levels in patients with IBD included tumor necrosis factor α, IL-1Ra, and growth related oncogene. The serum levels of IL-17a, sCD40L, Platelet-derived Growth Factor-AA, Macrophage-Derived Chemokine (MDC), and IFNγ were lower in patients with IBD than in the healthy control patients, suggesting a unidirectional flow of cytokines toward the site of the inflammation.^18^ However, none of the cytokines differed significantly in concentration between the patients with UC and patients with CD. Spearman’s correlation analysis revealed a positive correlation of calprotectin and S100A12 with IP-10, which may reflect the important role of neutrophils in IBD. Calprotectin and S100A12 were negatively correlated with MDC, and lactoferrin was positively correlated with growth related oncogene and negatively correlated with MDC and IFNγ.

Altogether, the data confirmed that the low specificity of fecal biomarkers and the heterogeneity of circulating cytokines do not allow one to distinguish between UC and CD.

### Microbiota Analysis by 16S rRNA Sequencing

**FIGURE 3.**
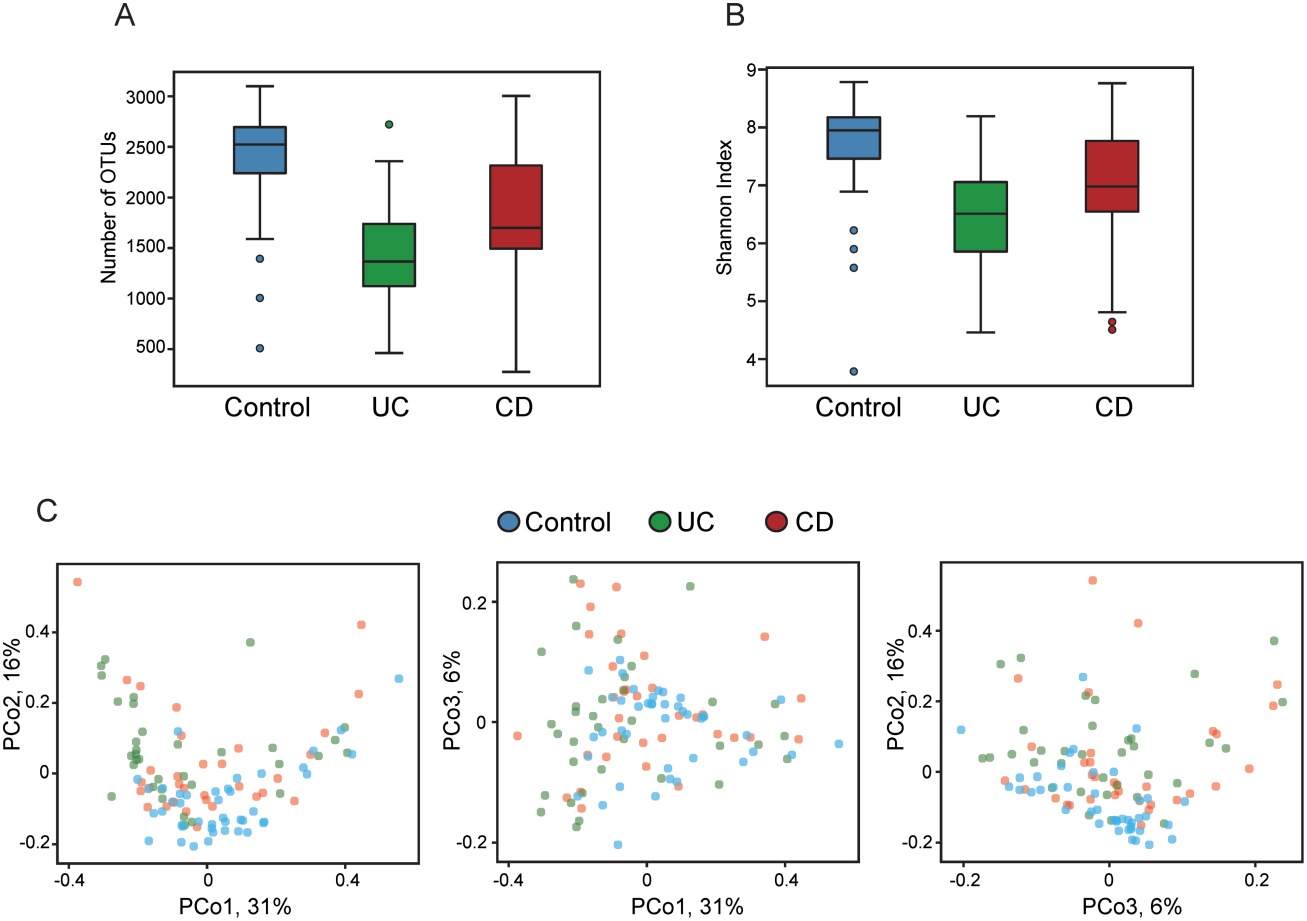
Taxonomic analysis of 16S data. A, Number of OTUs per group. B, Shannon diversity index calculated for the 3 study groups. C, Principal coordinate analysis of the weighted UniFrac distances matrix calculated on the basis of OTU counts. All pairwise combinations of the first 3 principal coordinates are shown. Each dot represents 1 sample. Samples colored on the basis of study group.

To analyze the metagenomic profile of the patients in this study, we first performed amplicon sequencing of the 16S rRNA gene in fecal DNA extracts. The number of sequencing reads obtained from the initial 109 fecal samples ranged from 38,000 to 164,000, and the numbers of operational taxonomic units (OTUs) were in the following range for each group: 508 to 3100 (control group), 461 to 2721 (UC group), and 274 to 3004 (CD group) (Fig. 3A).

The Shannon index was calculated using the abundances obtained across all reported OTUs for these 3 groups. Fig. 3B shows that no significant differences were observed in the Shannon index among the groups, with only those in the UC group exhibiting a trend toward lower diversity (not statistically significant) than those in the other groups. Variability was higher in the IBD groups than in the control group; this difference was probably attributable to the medications and the consequent confounding effect that they may have exerted.

Distances between samples were assessed using the weighted UniFrac metric. Principal coordinate analysis of the distance matrix thus obtained showed weak clustering by IBD type, with the first 3 principal coordinates capturing 53% of the variation in the input data (Fig. 3C).

### Microbiota Analysis by Shotgun Metagenomics

**FIGURE 4.**
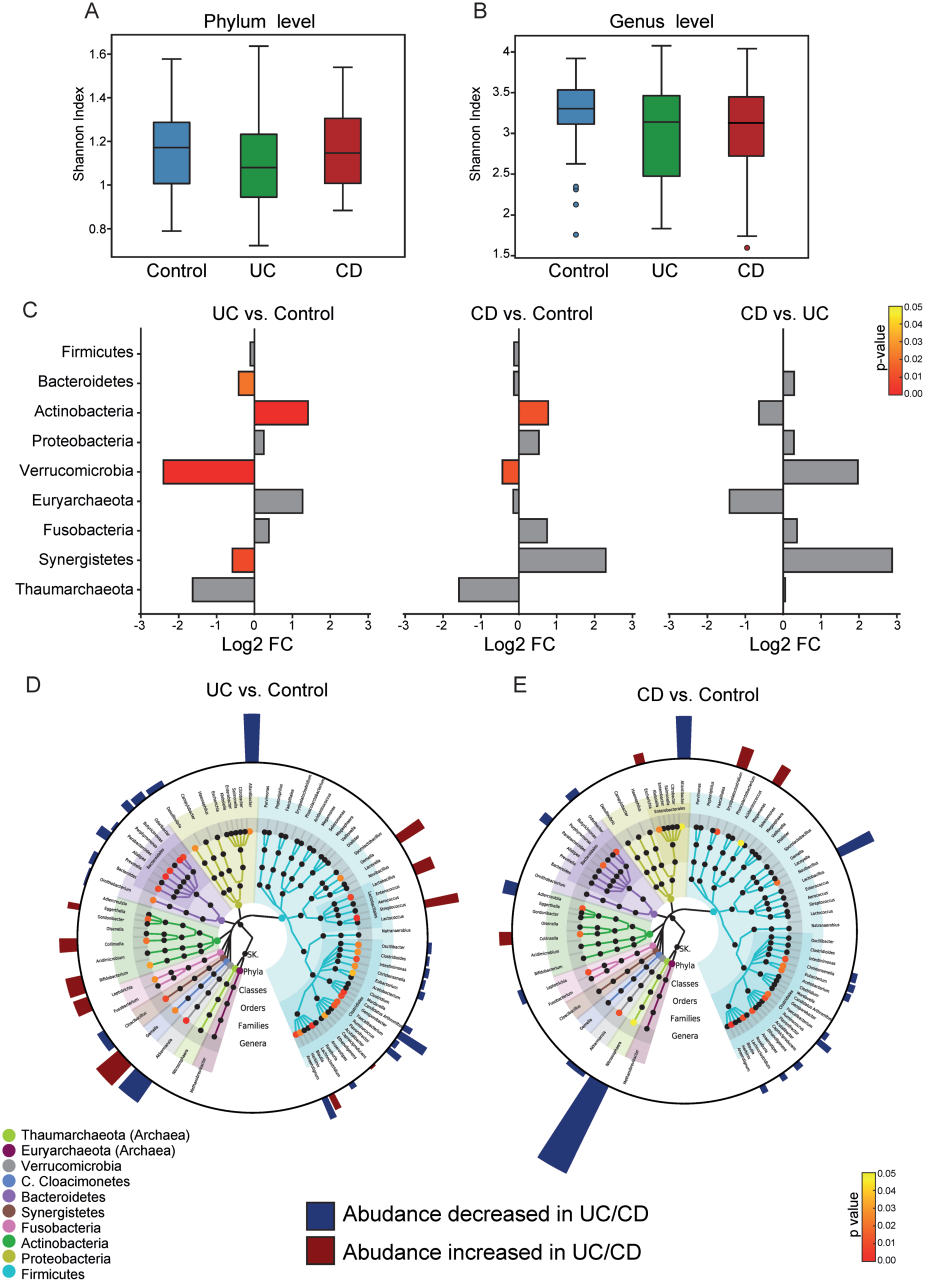
Taxonomic analysis of WGS data. A, Shannon diversity index calculated at phylum level for the 3 study groups. B, Shannon diversity index calculated at genus level for the 3 study groups. C, Bar plot of log_2_ ratio of taxonomy abundances at phylum level in 2 different study groups. Analysis performed for phyla with relative abundance ≥0.1 in at least 1 sample. The combination of 2 study groups for which the analysis was performed is shown at top of panel (patients with UC vs control patients: left panel; patients with CD vs control patients: central panel; patients with CD vs patients with UC: right panel). Negative value indicates decrease in abundance in first study group relative to second study group. Positive value indicates increase in abundance in first study group relative to second study group. Color of each bar represents *P* value for the null hypothesis that distribution of abundance for patients in both study groups is equal. Bars colored in gray for *P* values >0.05. D, Log_2_ ratio of taxonomy abundances at the genera level in UC and control patient groups shown as a bar plot associated with phylogenetic tree. Analysis performed for genera with relative abundance ≥0.1 in at least 1 sample. Height of bar in outer ring of taxonomy tree is proportional to the log_2_-change ratio of abundance of the particular genera in patients with UC and control patients. Increase/decrease in abundance is indicated by bar color. Bar thickness adjusted to give a better balance to the picture and has no analytical meaning. Log_2_-change ratio is reported only for genera with *P* values ≤0.05 for the null hypothesis that distribution of abundance in patients with UC and control patients is equal. Color of the terminal nodes represents *P* value (only if ≤0.05). Color of the tree branches indicates bacterial phylum. E, Log_2_ ratio of taxonomy abundances at the genera level in patients with CD and control patient groups shown as a bar plot associated with phylogenetic tree. Panel has same indication as panel D.

To complement the 16S-based analysis, we performed shotgun metagenomics analysis, a technique that offers increased resolution, enabling more specific taxonomic classification of sequences and providing direct assessment of the functional attributes of the microbiome.^19^ Analysis of the Shannon index at the phylum and genus levels revealed only a weak and nonsignificant decrease in the diversity of microbiota in the IBD cohort (Figs. 4A and B). Analysis of the differential abundance of phyla and genera was restricted to taxa whose abundance was >1% (Figs. 4C-E) in at least 1 sample. The UC and CD groups exhibited similar trends at the phylum level relative to the control group (Fig. 4C). Whereas the abundance of Firmicutes (not significant), Bacteroidetes (significant only in the UC group relative to the control group), and Verrucomicrobia was decreased, that of aerotolerant taxa such as Actinobacteria, Proteobacteria (not significant), and Fusobacteria (significant only in the CD group relative to the control group) was increased (Fig. 4C). Two archaea phyla were identified: Euryarchaeota, a methanogen archaeon, tended to be higher in abundance in patients with UC and significantly lower in patients with CD, and Thaumarchaeota exhibited a consistently, although not significantly, lower abundance in the patients with UC and those with CD than in the control patients (Fig. 4C).

Analysis of differential abundance at the genus level mirrored the fluctuations observed in the phyla but with some specificity (Figs. 4D and E; Supplementary Fig. 4). For example, among the Firmicutes, genera belonging to the order Clostridiales (both the Clostridiaceae and Lachnospiraceae families)—such as *Faecalibacterium, Eubacterium, Ruminococcus,* and *Roseburia*—were consistently lower in both patients with UC and those with CD than in control patients. In contrast, the abundance of *Lactobacillus* and *Streptococcus,* belonging to the order Lactobacillales, was higher in patients with IBD but significantly higher only in those with UC (Supplementary Fig. 4). The decreased abundance of *Bacteroidetes* in patients with IBD was probably driven by the decrease in some of the most abundant taxa, such as Bacteroides, Prevotella, Alistipes, and Parabacteroides. Interestingly, less abundant but still important SCFA producers such as *Butyricimonas* and *Odoribacter* were also significantly decreased in both IBD groups (Figs. 4D and E; Supplementary Fig. 4). The greatest decrease in abundance in both IBD groups was observed for *Akkermansia* (phylum Verrucomicrobia), key propionate-producing and mucin-degrading microorganisms.^20^

Finally, *Escherichia, Haemophilus, Bifidobacterium,* and *Collinsella* were the main genera contributing to the increased abundance of Proteobacteria and Actinobacteria (Figs. 4C-E; Supplementary Fig. 4). *Cloacibacillus,* belonging to the phylum Synergistetes, was increased (although not significantly) only in patients with CD and showed a significantly decreased abundance in patients with UC. The observed archaea dysregulation was probably a result of the changes in the abundance of *Methanobrevibacter* (phylum Euryarchaeota) and *Nitrososphaera* (phylum Thaumarchaeota) (Figs. 4D-E; Supplementary Fig. 4). Overall, the data showed a profound dysbiosis in IBD. Comparisons at the phylum and genus levels did not reveal significant differences between patients with CD and UC, suggesting that alternative approaches are required.

### SCFA Abundance

**FIGURE 5.**
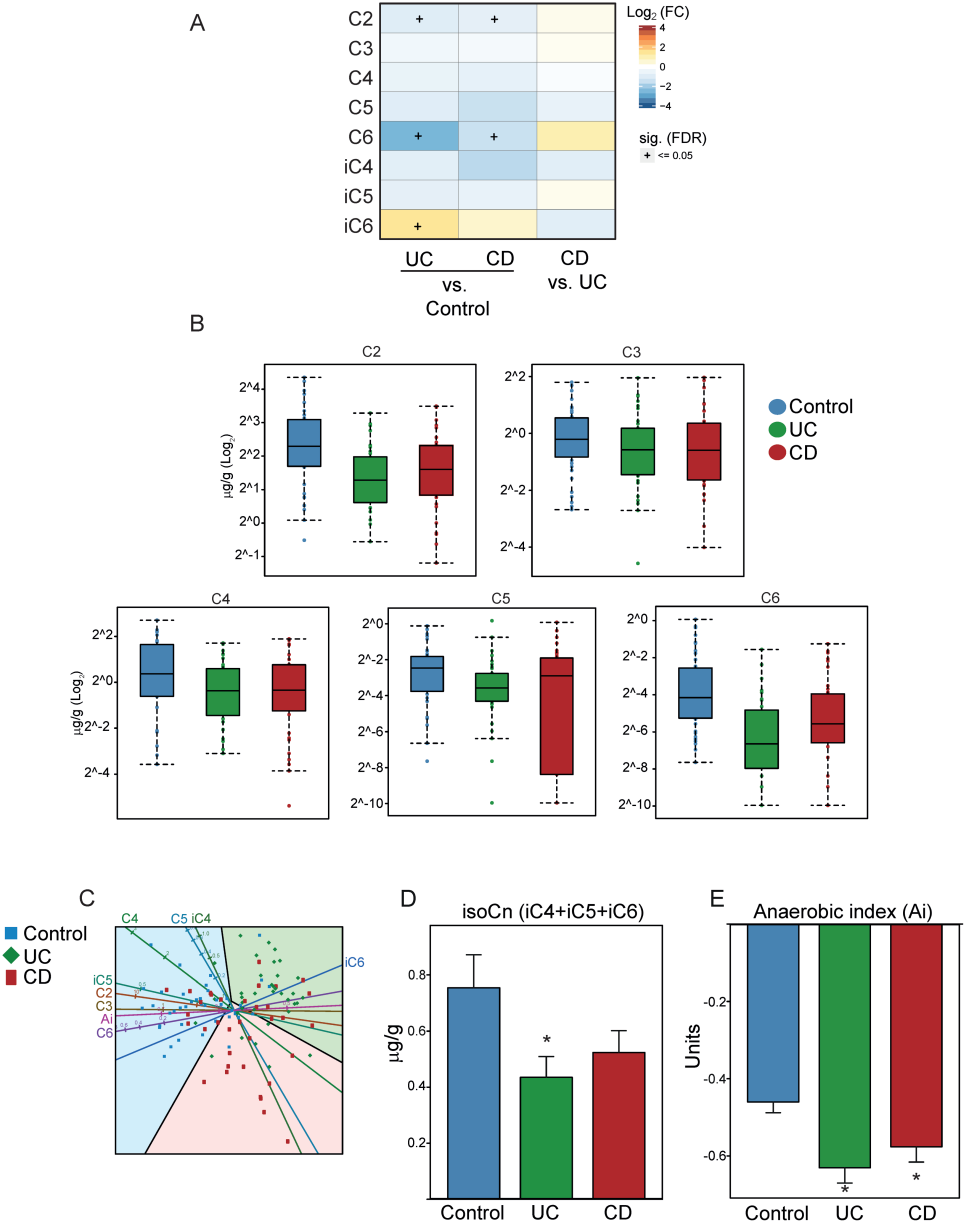
Differential abundance of fecal SCFAs. A, Heatmap representing abundance of differential SCFAs in patients with UC and CD relative to healthy control patients or each other. Differential abundance displayed using an intensity-dependent color map and complemented by statistical significance (+ = adjusted [false discovery rate] *P* value ≤0.05). B, Boxplot representing selected SCFA abundance in the 3 study groups plotted on a log_2_ scale. Dots superposed on resulting boxplots to better depict data distribution. A few points are not visible because they may be covered by boxplots. Sample medians depicted by arm using black lines inside boxes. Lower and upper box limits correspond to first and third quartiles of sample values. The whiskers extend boxes on both sides by an amount proportional to data variability, because this is quantified through the interquartile range. C, Canonical variate analysis biplots. Patients (points) and SCFAs (axes) simultaneously projected on 2-dimensional canonical axes (CA1 and CA2). Thick solid lines colored by group depict the 0.95-bags for each group, reflecting the deepest 95% core of data for a given group. Prediction regions for each study arm also depicted using study arm colors. D, Bar charts for the mean of total isoCn (iC4, iC5, and iC6) concentration by study arm. Error bars illustrate standard error of the mean and asterisks, when present, depict statistically significant differences (*P value ≤0.05). E, Bar charts for mean Ai by study arm. Error bars illustrate standard error of the mean and asterisk, when present, depicts statistically significant differences (*P value ≤0.05).

The main SCFA-producing bacteria in the gut are obligate anaerobes and include genera belonging to the most abundant phyla listed in Fig. 4C, many of which were significantly decreased in abundance in both patients with UC and patients with CD (eg, *Faecalibacterium, Eubacterium, Ruminococcus, Roseburia, Bacteroides, Odoribacter,* and *Akkermansia*). To better understand how bacterial metabolic products are affected by IBD, we measured the SCFA levels in fecal samples by gas-liquid chromatography (Figs. 5A, B). The levels of almost all SCFA species were lower in patients with UC and those with CD than in healthy control patients, but only acetate (C2) and caproate (C6) levels were significantly lower. This trend was confirmed by canonical variate analysis, which extracted the SCFA profile (reflected by the canonical variate components) that best separated the study groups (Fig. 5C). The lower concentrations of SCFAs allowed a clear separation among these 3 study groups on the first canonical axis. Branched-chain fatty acids (BCFAs) such as isobutyrate (iC4), isovalerate (iC5), and isocaproate (iC6) were also quantified (Figs. 5A and D; Supplementary Fig. 5). Both patients with UC and patients with CD showed decreased levels of total BCFAs (Fig. 5D) and 2 main species, iC4 and iC5 (Supplementary Fig. 5), and, contrarily, increased levels of iC6.

The SCFAs are the end products of anaerobic colonic bacterial fermentation of nondigestible carbohydrates or proteins. Thus, we measured anaerobic index (Ai) as the sum of the concentrations (C) of reduced acids (C3 + C4) to a less reduced acid (C2) (eg, [C3 + C4]/C2). The value of the Ai therefore represented the oxidation-reduction (redox) potential of the intestinal milieu. In patients with UC and CD, the Ai shifted to more negative values (Fig. 5E), a condition compatible with the overgrowth of facultative anaerobic bacteria.^21^

To identify the bacteria associated with fecal SCFA levels in patients with IBD, we performed correlation analyses between the abundance of bacterial genera and fecal SCFAs (Supplementary Fig. 6). Surprisingly, only a few genera were significantly correlated with specific SCFAs. The abundance of *Megasphaera*—which can reportedly produce several SCFAs, such as acetate, propionate, butyrate, and valerate^22^—was positively correlated with all of these SCFAs but reached statistical significance only for valerate. *Megasphaera* abundance did not vary among the 3 study groups (Supplementary Fig. 4). The genus *Candidatus Cloacimonas* was increased in abundance in both IBD groups. The abundance of the main bacteria known to be involved in SCFA production, including *Faecalibacterium, Eubacterium, Alistipes, Roseburia, Ruminococcus, Bacteroides,* and *Prevotella,* was positively correlated, although not always significantly, with most of the SCFA species analyzed. Only *Ruminococcus, Bacteroides,* and *Prevotella* abundances reached statistical significance for caproate, isocaproate, and propionate levels, respectively. *Akkermansia,* although involved in the production of both acetate and propionate and significantly reduced in abundance in both patients with UC and patients with CD, had a negative but nonsignificant correlation with acetate, propionate, and butyrate levels, similar to the conditions observed in the gut of patients with psoriatic arthritis.^23^*Atlantibacter* (previously known as *Escherichia hermannii*),^24^ which is reduced in abundance in both UC and CD, was the only genus that exhibited a positive and statistically significant correlation with more than 1 species of SCFA. Finally, *Adlercreutzia,* recently associated with pediatric IBD,^25^ was positively associated with isovaleric acid.

Overall, these observations support the hypothesis that the dysbiosis phenotype associated with IBD is characterized by the reduced abundance of multiple SCFA-producing bacterial species, leading to reduced levels of anti-inflammatory SCFAs in feces.

### Microbial Pathway Abundance

We calculated the pathway abundance (Supplementary Fig. 7) for biochemical pathways and compared the groups by considering the log_2_ ratio of the means. There were no statistically significant differences between patients with UC and patients with CD, but the 2 groups differed from the control patients. In patients with CD, only the L-lysine biosynthesis pathway showed a significantly increased abundance; this trend was even more significant in patients with UC. Moreover, patients with UC exhibited significant changes in the abundance of multiple pathways, including overrepresentation of isoprene biosynthesis, which could be indicative of pathogenic activity.^26^ The closely linked mevalonate pathway, which was also overabundant in the UC group, is the only certain route for Gram-positive bacteria to produce isoprenoids. Among the highly overabundant pathways in UC, peptidoglycan biosynthesis overabundance may be indicative of Lactobacillaceae abundance.^27^

The changes in 2 pathways particularly relevant to IBD are those affecting fatty acid biosynthesis: pyruvate fermentation and 4-aminobutanoate degradation (Supplementary Fig. 7). The pyruvate fermentation pathway leads to the production of propanoate, a substance generally associated with anti-inflammatory action in the gut. This pathway was barely affected in the patients with CD but was heavily underrepresented in those with UC. Likewise, the concentrations of proteins responsible for 4-aminobutanoate degradation were lower in patients with UC. This is particularly relevant because butyrate, like propanoate, has been shown to exert anti-inflammatory effects on the gut wall, together with the other physiological effects associated with SCFAs. The third pathway that was underrepresented in the patients with UC is related to the gamma-aminobutyric acid shunt and may be indicative of the reduced abundance of *Bacteroides* and *Parabacteroides* in patients with UC (Fig. 4D). Together with *Escherichia*, these are the main genera that actively express proteins involved in gamma-aminobutyric acid–producing pathways.^28^ Finally, a global increase in amino acid biosynthesis (L-lysine, L-arginine, and L-ornithine), glycolysis, and purine and nicotinammide adenine dinucleotide salvage was observed, indicating a major imbalance in energy metabolism in patients with IBD.

## DISCUSSION

The gut microbiota of people in Western countries have been extensively investigated during the last number of years, resulting in an increasing volume of data and knowledge. Because of the rising IBD prevalence in newly industrialized countries such as those in Asia, the Middle East, and South America,^1^ there is greater interest in elucidating the gut microbiota of these populations.^10^

The current study is the first to perform a cross-sectional full characterization of IBD-associated biomarkers and gut microbiota profile and functions in a Kazan population of patients with IBD. Because of its geographic location, Kazan has historically been considered a melting pot of Eastern and Western cultures. The Kazan region is inhabited by a total of 173 different ethnicities (registered during the 2010 census), 8 of which (Russians, Tartars, Chuvash, Ukrainians, Mari, Mordvins, Bashkirs, and Udmurt) had populations of >10,000 people.^29^ This peculiar condition makes Kazan particularly interesting as one of the most multinational and multicultural territories in the Russian Federation, nowadays representing a perfect model of a modern, multiethnic, and multilifestyle country in Eastern Europe.

Our first-tier analysis validated and characterized the clinical condition of the study groups. The findings of a classical clinical evaluation of the patients with IBD and the healthy control patients (eg, disease history review, endoscopy, and histopathological assessment) were supported by the results of a full panel of blood and fecal inflammatory biomarkers. Disease activity in both patients with UC and patients with CD was confirmed by the increased levels of all fecal biomarkers analyzed, which showed a clear correlation with the disease activity state, confirming previous observations.^16, 17^ Interestingly, the level of inflammatory biomarkers was associated with the therapeutic interventions. Indeed, patients treated simultaneously using 2 or 3 drugs showed a higher concentration of the analyzed biomarkers if compared with patients treated using only 1 drug. These data represent the association between treatment and disease activity. Thus, patients with higher disease activity and levels of related biomarkers were under a stronger treatment regimen (ie, they received multiple medications simultaneously). Nevertheless, none of these biomarkers showed specificity for UC or CD, which confirmed that they can be used to distinguish between IBD and a healthy status but not to discriminate between IBD subtypes.

Furthermore, in this study we analyzed common serological IBD biomarkers such as ASCA and perinuclear anti-neutrophil cytoplasmic antibodies; however, only a few patients with IBD tested positive for these markers, which confirmed their low sensitivity^30^ and limitation for use in IBD screening in the general population. However, the high specificity observed for human ASCA IgA in patients with CD may support its usefulness in differentiating between CD and UC.

Unfortunately, serum cytokine quantification also failed to identify significant differences between patients with UC and patients with CD. Of the 42 cytokines included in our analysis, only 15 were detectable in a consistent number of patients, and their differential levels confirmed that systemic cytokine levels are not effective for predicting or diagnosing IBD.^18^ Only the chemokines Regulated upon Activation, Normal T Cell Expressed and Presumably Secreted and IP-10 and the cytokine IFNα2 were significantly higher in concentration in patients with IBD, whereas the other cytokines detected were lower in concentration. We surmise that most of these cytokines are limited to tissue sites of inflammation or have relatively short half-lives and are therefore lower in concentration or not detectable in serum.

To elucidate the dominant dysbiosis pattern in the study population, we profiled the metagenome by both 16S and shotgun sequencing. Although 16S amplicon sequencing remains a standard procedure in taxonomic profiling of metagenomics data, an increasing number of studies have shown biases associated with this method.^31^ Moreover, shotgun metagenomics analysis offers increased resolution in taxonomic and functional classification. Therefore, we applied computational analyses of metagenomics data solely to the shotgun sequencing reads.

It is broadly accepted that microbiota diversity decreases in patients with active IBD. We observed a trend toward a decrease in the number of OTUs and Shannon index values upon 16S sequencing; this trend was only partially confirmed at the genus level upon WGS. These observations are in line with previous reports that have explained these results as the consequence of confounders such as medication or the site of inflammation.^10^ Furthermore, previous conclusions on the decrease in biodiversity were based on studies in Western countries and thus may not apply to other populations, such as in Kazan or, as previously shown, China.^10^

Similar to previous observations, our findings highlight the classical IBD dysbiotic feature at the phylum level: increased abundance of Proteobacteria, Actinobacteria, and Fusobacteria and decreased abundance of Firmicutes, Bacteroidetes, and Verrucomicrobia.^6^

The observed phylum-level fluctuations were mirrored by the differential abundance of specific genera. In agreement with previous findings,^32^ we found that *Escherichia* (phylum Proteobacteria) abundance was increased only in patients with CD, which may indicate the presence of *E. coli*, known to be a major pathobiont in IBD development. An increase in Actinobacteria abundance was more evident in patients with UC and was driven by the increased abundance of Collinsella and Bifidobacterium. The latter is known to improve the clinical symptoms of IBD and hence is a common supplement in probiotics. This ostensible inconsistency was actually similar to that in previous findings by Wang and colleagues.^33^ It is worth noting that Bifidobacterium species are phylogenetically diverse, with more than 50 documented species to date.^34^ For example, *B. longum* has been reported to shift immune responses toward a proinflammatory or regulatory profile and thus modulate their effects.^35^ Thus, we cannot exclude that the increased *Bifidobacterium* genera detected in our study correspond to species that are considered proinflammatory by others.

The most striking IBD-associated effect was observed in SCFA-producing bacteria,^9^ which mainly belonged to the Firmicutes, Bacteroidetes, and Verrucomicrobia phyla. Thus, the abundance of genera such as *Faecalibacterium, Ruminococcus, Roseburia, Eubacterium, Butyricimonas, Odoribacter,* and *Akkermansia* was lower in patients with IBD than in the control patients. These observations were confirmed by direct SCFA measurement and functional classification of the detected genera. The levels of almost all SCFA species detected in the feces of patients with UC and with CD were lower than those in the healthy control patients. Similarly, 2 pathways involved in fatty acid biosynthesis, pyruvate fermentation and 4-aminobutanoate degradatio seemed to be underrepresented in the IBD groups, although the decrease was only statistically significant in patients with UC.

The physiological role of SCFAs has been researched and reviewed extensively.^9^ Our data also showed a decrease in the levels of 2 BCFAs: isobutyrate and isovalerate. The BCFAs can be oxidized when butyrate is not available, and isobutyrate-forming species may account for up to 40% of total intestinal anaerobes.^36^ Isocaproate was the only fatty acid with increased levels in both patients with UC and patients with CD, although the increase reached statistical significance only in UC. Not much is known about isocaproate, and as a product of protein fermentation it may also be toxic to the intestine. We can speculate that isocaproate may be responsible for some of the mucosal damage observed in IBD, although this hypothesis would require further analysis.

Apart from SCFAs, carbohydrate and protein fermentation also generates hydrogen (H_2_) and carbon dioxide. To counteract oxidative stress in the intestine, H_2_ production and disposal in the intestinal lumen should be closely balanced. Growing evidence shows that regulation of H_2_ is important in IBD, colorectal cancer, gastrointestinal infections, obesity, and associated metabolic disorders.^37^ In healthy patients, the adverse health effects related to an imbalance in H_2_ metabolism are counteracted by a stable symbiosis between fermentative (SCFA-producing and H_2_-releasing) and hydrogenotrophic (H_2_-consuming) microbes.^38^ The human gut contains 3 major groups of hydrogenotrophs—methanogens, sulfate-reducing bacteria, and acetogens.

In our study, we found that IBD status affects both H_2_-releasing and hydrogenotrophic microbes. Indeed, *Ruminococcus,* an acetogenic genus, was decreased in patients with IBD. *Methanobrevibacter,* a euryarchaeotic methanogenic genus, was overabundant in patients with UC, although not to a statistically significant extent. Finally, *Veillonella, Streptoccocus, Leptotrichia, and Desulfovibrio,* all sulfate-reducing bacteria, were also affected in both patients with UC and patients with CD. Although *Desulfovibrio* was less abundant in both patients with UC (significantly) and patients with CD, *Veillonella, Streptoccocus,* and *Leptotrichia* were overabundant in patients with UC. Thus, our data highlight an imbalance in all types of microbes involved in H_2_ metabolism, and this imbalance was confirmed by the change in the Ai, which in turn mirrors the redox status of the intestinal lumen. The Ai has been previously defined as the ratio of the sum of the concentrations of more reduced to less reduced acids, eg, (propionic + butyric)/acetic.^39, 40^ In organic chemistry, the redox balance is affected by both oxygen and H_2_ transfer and availability. A deviation toward a more negative value, such as in patients with IBD, indicates an increase of the numerator of the previous fraction ([propionic + butyric]/acetic), thus meaning an increase in the more reduced species. This condition reflects a shift in the redox balance of products, with an increase in oxygen (a condition compatible with facultative anaerobic bacteria^21^) and a decrease in H_2_ availability.^41^

Moreover, in our study, functional analysis of the gut microbiota showed a dysregulation of microbial pathways such as nicotinammide adenine dinucleotide salvage and glycolysis, both overrepresented in IBD and connected to H_2_ metabolism.

**FIGURE 6.**
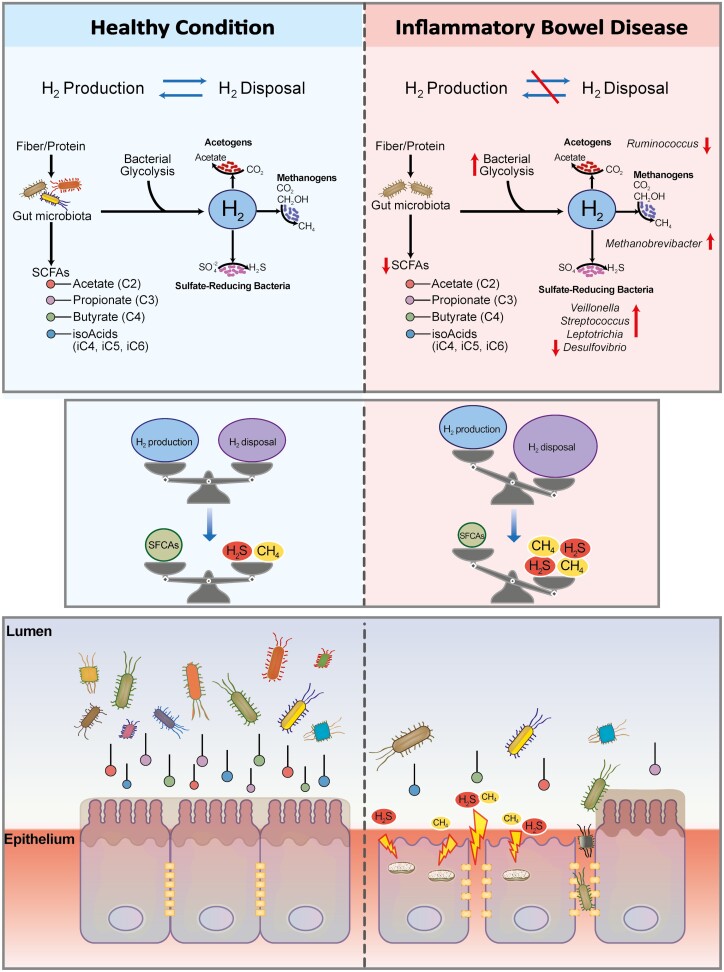
Schematic representation of possible microbiota-related metabolic changes. The H_2_ production and disposal in the intestinal lumen are tightly balanced to avoid uncontrolled increase in oxidative stress. Carbohydrate and protein fermentation by SCFA-producing microbes and microbial glycolysis contribute to H_2_ production, and methanogens, sulfate-reducing bacteria, and acetogens are the 3 major groups of H_2_-consuming microbes. In a healthy condition (top left panel), H_2_-releasing and H_2_-consuming bacteria coexist in equilibrium. In patients with IBD, dysbiosis affects most taxa belonging to these 2 groups, disrupting this balance and causing an increase in production of harmful products such as H_2_S and methane.

The affected abundance of sulfate-reducing bacteria in the patients with IBD in our study may lead to speculations on the potential cascading effects on hydrogen sulfide (H_2_S) concentrations in the gut lumen. The role of H_2_S in IBD development is supported by the finding that microbial H_2_S formation contributes to mucus degradation, opening up the intestinal barrier to toxic compounds and pathogens.^42^ In addition, H_2_S is linked to SCFAs, and its imbalance may result in the exacerbation of intestinal inflammation. Thus, H_2_S may increase colonocyte oxygenation by inhibiting β-oxidation^43^ and lead to the inhibition of obligate anaerobes that produce SCFAs. Furthermore, butyrate is known to activate the expression of genes encoding the host mitochondrial H_2_S detoxification pathway.^44^ Thus, a decrease in the abundance of butyrate-producing microbes may dampen host H_2_S defense systems and consequently increase the susceptibility of the intestine to further damage. Overall, metagenomics analysis allow researchers to speculate on the impairment of 2 connected processes in the present IBD cohort—SCFA and H_2_ metabolism—that may contribute to disease progression and activity (Fig. 6).

Although we had a relatively high number of samples for shotgun metagenomics sequencing, comprehensive metadata analysis, and serum and fecal inflammatory biomarker panels, our study has some limitations. First, this is a cross-sectional study involving patients with an established medical history and not an inception cohort. In this case, the main limitation is related to the possible confounding of the results by factors such as diet, disease state, and medication.

Second, although our endpoints included circulating cytokine levels, fecal SCFA levels, and metagenomics changes, we could not obtain a clear molecular signature for differentiating patients with UC from patients with CD. Although several attempts have been made to use dysbiotic features as a diagnostic tool or disease biomarker, the larger interindividual variation compared with interdisease differences precluded the identification of microbial constituents that could consistently distinguish between the 2 groups. A possible explanation comes from the high percentage of “dark matter” or “unknown” reads, which account for up to 60% of the entire sequenced DNA^45^ and may contain yet unknown information.

Third, based on the presented data, this study describes possible associations between gut dysbiosis and IBD and cannot prove causation, so the question of whether the observed dysbiosis is the cause or simply a consequence of inflammation remains to be answered. Turning epidemiological observations from correlation to causation has well-known limitations^46^ and highlights the needs of longitudinal studies and complementary preclinical approaches to reinforce the mechanistic insights behind the complex host–microbiota interaction. The recent efforts of the Integrative Human Microbiome Project^7^ are turned in this direction, highlighting the need to leverage a “multi-meta-omics” approach in such studies. The integration of taxonomic and functional microbiota profiles, together with dedicated mathematical and computational approaches, will be necessary to untangle these important points.

## CONCLUSIONS

In conclusion, we have presented a comprehensive analysis of several IBD-related biomarkers and the gut microbiota profile and functions of a unique Kazan population of patients with IBD and healthy patients. Our analyses have highlighted how IBD-related dysbiotic microbiota, which are generally mainly linked to SCFA imbalance, may affect other important metabolic pathways, such as H_2_ metabolism, that are critical for host physiology and disease development. Further -omics-based approaches are necessary to understand how the alterations in gut microbiota are reflected by the changes in the metabolic activity of microbes and how such fluctuations may impact health and disease in the human colon to eventually discover novel prophylactic or therapeutic options for IBD.

## Supplementary Material

izaa188_suppl_Supplementary_Material

izaa188_suppl_Supplementary_Figure_1

izaa188_suppl_Supplementary_Figure_2

izaa188_suppl_Supplementary_Figure_3

izaa188_suppl_Supplementary_Figure_4

izaa188_suppl_Supplementary_Figure_5

izaa188_suppl_Supplementary_Figure_6

izaa188_suppl_Supplementary_Figure_7

## Notes

Author contributions: All authors have made substantial contributions to the conception and design of the study, the acquisition and analysis of data, and the redaction and revision of the article and have approved the final version submitted. Lo Sasso contributed to the study design, interpreted the data, and wrote the article. Khachatryan performed bioinformatics analysis of the microbiome sequencing data and participated in article preparation. Kondylis was accountable for statistical analysis. Battey performed microbial pathway abundance analysis and participated in article preparation. Sierro and N. Ivanov supervised microbiota data analysis. Peitsch and Hoeng conceived the study and contributed to the study design and manuscript preparation. Laikov, Salafutdinov, and Romanova contributed to immunoferment analysis of inflammation markers. Siniagina, Khusnutdinova, and Grigoryeva contributed to metagenomic analysis of the intestinal microbiota. Boulygina and Markelova contributed to data analysis. Solovyeva, Chulpanova, and Kitaeva performed investigation of biomarkers in serum samples. Garanina and K. Ivanov evaluated cytokine levels in serum samples. Kletenkov contributed to statistical analysis. S. Abdulkhakov and R. Abdulkhakov recruited the patients and contributed to interpretation of the results and study design. Ardatskaya contributed to data analysis of the short-chain fatty acid results. Odintsova, Danilova, and Valeeva assisted the patients and collected metadata and blood.

Supported by: Philip Morris International is the sole source of funding and sponsor of this research.

Conflicts of interest: Lo Sasso, Khachatryan, Kondylis, Battey, Sierro, N. Ivanov, Peitsch, and Hoeng are employees of Philip Morris International. S. Abdulkhakov, Danilova, Grigoryeva, Markelova, Vasiliev, Boulygina, Solovyeva, Garanina, Kitaeva, K. Ivanov, Chulpanova, and Kletenkov are employees of the Kazan Federal University. Valeeva and R. Abdulkhakov are employees of the Kazan State Medical University. Odintsova is an employee of the Republican Clinical Hospital of Tatarstan Republic. Ardatskaya is an employee of the Central State Medical Academy of the Administrative Department of the President of the Russian Federation.
