# Supplementary material for: Inflammatory Bowel Disease–Associated Changes in the Gut: Focus on Kazan Patients
Source: Inflamm Bowel Dis. 2020 Aug 7;27(3):418–33. doi: 10.1093/ibd/izaa188 (PMC7885336; doi:10.1093/ibd/izaa188)
Supplement: izaa188_suppl_Supplementary_Material [file izaa188_suppl_supplementary_material.docx]

# **Supplementary Material**

**Inflammatory Bowel Disease associated changes in the gut: focus on Kazan patients.**

Giuseppe Lo Sasso^a*#^ PhD, Lusine Khachatryan^a#^ PhD, Athanasios Kondylis^a#^ PhD, James N.D. Battey^a^ PhD, Nicolas Sierro^a^ PhD, Natalia A. Danilova^b^ MD, PhD, Tatiana V. Grigoryeva^b^ PhD, Maria I. Markelova^b^ MSc , Dilyara R. Khusnutdinova^b^ MSc, Alexander V. Laikov^b^ MSc, Ilnur I. Salafutdinov^b^ PhD, Yulia D. Romanova^b^ PhD, Mariia N. Siniagina^b^ MSc, Ilya Yu. Vasiliev^b^ MSc, Eugenia A. Boulygina^b^ MSc, Valeriya V. Solovyeva^b^ PhD, Ekaterina E. Garanina^b^ PhD, Kristina V. Kitaeva^b^ MSc, Konstantin Y. Ivanov^b^ MSc, Darja S. Chulpanova^b^ MSc, Konstantin S. Kletenkov^b^ PhD, Alina R. Valeeva^c^ MD, Alfiya Kh. Odintsova^d^ MD, PhD, Maria D. Ardatskaya^e^ MD, PhD, Doc Sci, Rustam A. Abdulkhakov^c^ MD, PhD, Doc Sci, Nikolai V. Ivanov^a^ PhD, Manuel C. Peitsch^a^ PhD, Julia Hoeng^a^ PhD, and Sayar R. Abdulkhakov^b^ MD, PhD^*^.

*^a^PMI R&D, Philip Morris Products S.A., CH-2000 Neuchâtel, Switzerland*

*^b^Kazan Federal University, Institute of Fundamental Medicine and Biology, 420111 Kazan, Tatarstan, Russian federation*

*^c^Kazan State Medical University, 420012 Kazan, Tatarstan, Russian federation*

*^d^Republican Clinical Hospital of Tatarstan Republic, 420012 Kazan, Tatarstan, Russian federation*

*^e^Central State Medical Academy of Administrative Department of the President of the Russian Federation, 121359 Moscow, Russian Federation*

^#^Equally contributed

***Corresponding Authors**:

Giuseppe Lo Sasso, PhD, Scientist – Metabolic Diseases, PMI R&D, Philip Morris Products S.A. Quai Jeanrenaud 5, 2000 Neuchatel, Switzerland. [Giuseppe.Losasso@pmi.com](mailto:Giuseppe.Losasso@pmi.com). Phone: +41 (58) 242 2931.

Sayar R. Abdulkhakov, MD, PhD – Kazan Federal University, Institute of Fundamental Medicine and Biology, 420008, Kremlyovskaya str., 18, Kazan, Russian federation. [sayarabdul@yandex.ru](mailto:sayarabdul@yandex.ru). Phone: +7917 9342192.

**Funding**

Philip Morris International is the sole source of funding and sponsor of this research.

**Disclosure of interest**: Lo Sasso G., Khachatryan L., Kondylis A., Battey J.N.D, Sierro N., Ivanov N.V., Peitsch M.C., and Hoeng J. are employees of Philip Morris International. Abdulkhakov S.R., Danilova N.A, Grigoryeva T.V., Markelova M.I., Vasiliev I.Y., Boulygina E.A., Solovyeva V.V., Garanina E.E., Kitaeva K.V., Ivanov K.Y., Chulpanova D.S., and Kletenkov K.S. are employees of the Kazan Federal University. Valeeva A.R. and Abdulkhakov R.A. are employees of the Kazan State Medical University; Odintsova A.K. is an employee of the Republican Clinical Hospital of Tatarstan Republic; Ardatskaya M.D. is an employee of the Central State Medical Academy of Administrative Department of the President of the Russian Federation.

**Article Summary:**

We present a comprehensive analysis of IBD-related biomarkers and gut microbiota of a unique population of IBD and healthy subjects from Kazan. Our analyses highlighted how IBD-related dysbiosis might affect important metabolic pathways, such as H_2_-metabolism, critical for disease development.

# **Supplementary Methods**

### *Enzyme-linked immunosorbent assay (ELISA)*

Serum samples from patients with Crohn's disease (n = 41) or ulcerative colitis (n = 43) and healthy controls (n = 42) were analyzed by ELISA by using the following commercial kits in accordance with the manufacturers’ protocols: anti-MPO pANCA ELISA (#ORG519, Orgentec Diagnostika GmbH); anti-PR3 cANCA ELISA (#ORG518, Orgentec); human anti-*Saccharomyces cerevisiae* antibody (IgA) ELISA kit (#MBS705423, MyBioSource); human anti-*S. cerevisiae* antibody (IgG) ELISA kit (#MBS704395, MyBioSource); and human lipopolysaccharides ELISA kit (#MBS266722, MyBioSource).

### *Cytokine profile in serum samples*

Cytokine production in serum was assessed by multiplex analysis by using the MILLIPLEX MAP Human Cytokine/Chemokine Magnetic Bead Panel - Immunology Multiplex Assay (HCYTOMAG-60k-41 PX, MilliporeSigma) in accordance with the manufacturer’s protocol. The kit measures the levels of the following analytes: sCD40L, EGF, FGF-2, Flt-3 ligand, fractalkine, G-CSF, GM-CSF, GRO, IFN-α2, IFN-γ, IL-1α, IL-1β, IL-1ra, IL-2, IL-3, IL-4, IL-5, IL-6, IL-7, IL-8, IL-9, IL-10, IL-12 (p40), IL-12 (p70), IL-13, IL-15, IL-17A, IP-10, MCP-1, MCP-3, MDC (CCL22), MIP-1α, MIP-1β, PDGF-AB/BB, RANTES, TGF-α, TNF-α, TNF-β, VEGF, Eotaxin/CCL11, and PDGF-AA. Serial dilutions of standards were prepared at concentrations of 2000, 400, 80, 16, and 3.2 pg/mL. Aliquots (200 μL) of wash buffer were incubated at room temperature for 10 min. After washing, the standards and controls were added in duplicate to the appropriate plate wells, along with the serum matrix solution, 25 μL of assay buffer, and mixed magnetic beads. The plates were sealed, placed on a shaker, and incubated overnight at 4℃.

The plates were washed three times with wash buffer and then incubated with detection antibodies for 1 h on a shaker at room temperature. Next, a streptavidin–phycoerythrin solution was added to each well. The plates were incubated for 30 min at room temperature on a shaker. Following a two-step wash, sheath fluid (150 μL per well) was added to each well. The beads were resuspended for 5 min on a plate shaker. Measurements were performed by using a Luminex 200 microplate reader (Austin, TX, USA; 100 µL, with 50 beads per bead set).

Cytokine concentrations were determined by using the MILLIPLEX Analyst software and by calibration modeling. Median fluorescence intensities were fitted on calibration standards by using a five-parameter logistic model. The median fluorescence intensities of the samples were then back-fitted to determine the cytokine concentrations (in picograms per milliliter).

### *Statistical analysis of molecular markers, cytokines, and SCFAs*

Data were analyzed by univariate and multivariate analyses. Univariate analysis was based on statistical modeling for each targeted marker, cytokine, and SCFA separately. Multivariate analysis used exploratory tools to assess the evidence overall. Canonical variate analysis biplots were drafted for biomarkers of inflammation, cytokines, and SCFA levels. These allowed efficient visualization of all data in two dimensions and highlighted the correlations between individual markers. Similar to principal component analysis, canonical variate analysis compacts information into two dimensions that explain most of the data variation and ensure the best discrimination among groups (represented here by disease status). Correlated markers move in groups in the same direction, which is associated with disease status. Multivariate analyses helped visualize the data variation in relation to the disease groups (arms), while univariate analyses helped construct statistical models that adjusted for demographic characteristics such as age and sex. For each targeted marker, cytokine, and SCFA, a linear statistical model was constructed on the log scale. The choice of the log scale was further justified by residual analyses of the resulting models and by standard data plotting techniques (boxplots were generated for all outputs), which showed a highly skewed distribution for all outcomes in the original scale for each study group (arm). Statistical comparisons among the study groups (ulcerative colitis, Crohn’s disease, and healthy control groups) were made by testing the linear model terms associated with the defined contrasts (ulcerative colitis vs. control, Crohn’s disease vs. control, and Crohn’s disease vs. ulcerative colitis). Fold-change estimates for these contrasts were illustrated in heatmaps, and statistical significance was based on the 5% significance level after adjusting for the false discovery rate in multiple testing.^1^

## ***Metagenomics analyses***

### *16S sequencing data analysis*

Sequences were processed as described by Meadows and colleagues.^2^ The average number of raw bacterial sequences per sample (assembled as described in ^3^) exceeded 74,000 (ranging from 37,187 to 164,854). Further analysis of sequences was performed by using the QIIME v. 1.9.1 software.^4^ The sequences were filtered for quality by using the following settings: a minimum 30 quality score over at least 75% of the sequence read; no ambiguous bases allowed; and 1 primer mismatch allowed. After quality filtering, truncation, chimera removal,^5^ and rarefaction, the average number of sequences per sample was 26,388. These remaining sequences were advanced to OTU picking at a 97% sequence similarity cutoff and to taxonomy assignment by using the GreenGenes database v. 13.8.^6^ To be included in the taxonomy, the OTU should have been assigned at least to 2 sequences. To characterize the richness and evenness of the bacterial community, alpha (within-sample) diversity indices were calculated by using Shannon metric. Similarities in microbial composition among the samples were evaluated by using beta diversity characteristics, which were estimated by using weighted Unifrac measures and further visualized by PCoA.

#### Functional profiling (pathway abundance)

Functional annotation for the WGS metagenomics data was generated by using Biobakery’s wmgx pipeline,^7^ beginning with the raw reads and using all default settings and default reference databases with the exception of the 16S database. The 16S database was generated by a text search for “16S” in the NCBI nucleotide database, selecting all sequences belonging to “Fungi”, “Protists”, “Bacteria”, “Archaea”, and “Viruses”, filtering for a length of 700–2000 base pairs, and saving these sequences to a single fasta format file. For further analysis, the “pathway abundance” file produced by the HUMAnN2 component of Biobakery was used.^8^

# **Supplementary Figures**

## **Supplementary figure 1.**

A. BMI versus age for the CD and control arms (left panel) and UC and control arms (right panel). Data are colored by disease severity, and linear regression lines are provided to depict the trend in BMI evolution across age for each study arm. B. Heatmap representing the differential abundance of fecal and serum biomarkers of inflammation in UC and CD subjects relative to healthy control subjects or each other. Differential abundance or correlation coefficients are displayed by using an intensity-dependent color map and complemented by their statistical significance (*adjusted [FDR] *p* value ≤0.05). Color codes and symbols: black, control; orange, mild; red, moderate.

## **Supplementary figure 2.**

A-B. Boxplots of inflammatory biomarkers’ abundance by disease status (control, UC, CD) and disease activity (mild, moderate). Classification to disease activity is based on the Mayo score for UC patients (A) and on the CDAI for CD patients (B). Sample medians are depicted by group using black thick lines inside the boxes. Lower and upper box limits correspond to the first and third quartiles of the sample values. Color codes and symbols: blue, control; light green, UC mild; dark green, UC moderate; light red, CD mild; dark red, CD moderate. UC, ulcerative colitis; CD, Crohn’s disease. * p<0.05, ** p<0.01, *** p<0.001 between UC/CD vs Control. ^#^ p<0.05, Mild vs Moderate.

## **Supplementary figure 3.**

A-B. Boxplot of inflammatory biomarkers’ abundance by disease status (blue for controls, greens for UC and reds for CD) and followed treatment medication (no treatment, single treatment, co-treatment. Sample medians are depicted by group using black thick lines inside the boxes. Lower and upper box limits correspond to the first and third quartiles of the sample values. Color codes and symbols: blue, control; very light green/red, UC/CD under no treatments; light green/red, UC/CD under single treatment (only one drug); dark green/red, UC/CD under co-treatments (combination of two or three drugs). Please refer to Supplementary table 2 for more information. UC, ulcerative colitis; CD, Crohn’s disease. * p<0.05, ** p<0.01, *** p<0.001 UC/CD vs Control.

C. Association plot between disease activity (in columns) and treatment medication (in rows) for both UC and CD patients. Positive (negative) associations between disease activity and medication is depicted by blue (red) circles. The magnitude of the association is proportional to the circle size and color scale (the latter is scaled to -1, 1 in a correlation-fashion).

## **Supplementary figure 4.**

The log2 ratio of taxonomy abundances at the genera level in two different study groups, shown as a bar plot. Analysis was performed for genera with relative abundance ≥0.1 in at least one sample. The combination of two study groups for which the analysis was performed is shown at the top of the figure. The negative value indicates the decrease in abundance in the first study group relative to the second study group. The positive value indicates the increase in abundance in the first study group relative to the second study group. The color of each bar represents the p value for the null hypothesis that the distribution of abundance for samples in both study groups are equal. The bars are colored in gray in case of p values >0.05.

## **Supplementary figure 5.**

Boxplot representing selected SCFA abundance in the three study groups, plotted on the log2 scale. From left to right: control, UC, and CD. Dots are superposed on the resulting boxplots to better depict the data distribution. Few points are not visible because they could be covered by the boxplots. Sample medians are depicted by arm by using black lines inside the boxes. Lower and upper box limits correspond to the first and third quartiles of the sample values. The whiskers extend the boxes on both sides by an amount proportional to the data variability, because this is quantified through the interquartile range. For statistical differences, refer to Figure 5A.

## **Supplementary figure 6.**

Correlation between genera and SCFAs as estimated via the correlation coefficients. Correlation coefficient values are color-coded from blue (negative correlation) to red (positive correlation). Genera are grouped according to their differential abundance in UC and CD subjects relative to the healthy control subjects. Stars are used to highlight correlation coefficients that significantly different from zero on the adjusted 5% significance level (*adjusted [FDR] p value ≤0.05).

## **Supplementary figure 7.**

Bacterial pathway abundances. Log2 fold changes in pathway abundance for each of the contrasts between CD, UC, and healthy control subjects. Only pathways with a statistically significant (adjusted p value <0.05) fold change >2 (>1 in log space) are included here. UC, ulcerative colitis; CD, Crohn’s disease.

# **Supplementary Tables**

**Supplementary table 1.** Inclusion/exclusion criteria for IBD and healthy subjects.

| **Inclusion criteria for IBD subjects** | **Exclusion criteria for IBD subjects** |
| --- | --- |
| ♂/♀ aged 18 to 60 years | Alcohol consumption (2 weeks prior to screening); regular smoking (6 months prior to screening) |
| UC/CD diagnosis established at least 3 months prior to screening by clinical and endoscopic evidence, corroborated by histopathological findings | Presence of concomitant diseases/conditions which can lead to sufficient changes in the gut microbiota (e.g., malabsorbtion syndrome, oncological pathology, and prior GI surgery) |
| Subjects with extensive colitis for >8 years or left-sided colitis for >12 years must have documented evidence that surveillance colonoscopy was performed within 12 months of their visit for sample collection | The subject has any identified congenital or acquired immunodeficiency |
|  | *C. diff* infection or clinically significant infections, including HIV, viral hepatitis, tuberculosis, pneumonia, and urinary tract infection |
|  | Extensive colonic resection, or ≥3 small bowel resections, or short bowel syndrome. Ileostomy or colostomy. History of tube feeding |
|  | The subject has a history or evidence of colonic mucosal dysplasia |
|  | Pregnancy or breastfeeding |
|  | Intake of pre- and probiotics within 3 months prior to the visit for biosample collection; antibiotics within 6 months prior to the visit for biosample collection |
| **Inclusion criteria for healthy volunteers** | **Exclusion criteria for healthy volunteers** |
| ♂/♀ aged 18 to 60 years | Alcohol consumption (2 weeks prior to screening); regular smoking (6 months prior to screening) |
| The subject have not donated whole blood or blood components within 3 months prior to screening | Presence of concomitant diseases/conditions which can lead to sufficient changes in the gut microbiota (e.g., malabsorbtion syndrome, oncological pathology, and prior GI surgery) |
|  | The subject has any identified congenital or acquired immunodeficiency |
|  | Presence of chronic decompensated cardiovascular, respiratory, liver, kidney, or endocrine diseases; infectious or parasitic diseases, incl. HIV, viral hepatitis, and tuberculosis |
|  | Presence of diarrhea (stool frequency more than 3 times a day) within at least 3 consecutive days during the last month |
|  | Intake of some medications (immunosupressants, cytostatics, steroids, or pre- and probiotics) within 3 months prior to screening; antibiotics within 6 months prior to screening |
|  | Pregnancy or breastfeeding |

**Supplementary table 2**. Therapeutic intervention.

|  | **Number of Subjects** | | **Group** |
| --- | --- | --- | --- |
| **Therapy** | **Crohn’s disease** | **Ulcerative colitis** |  |
| 5-ASA | 14 | 8 | Single Treatment |
| Steroids | 4 | 1 |  |
| Immunosupressor | 0 | 6 |  |
| Biologics | 0 | 2 |  |
| 5-ASA+Steroids | 6 | 0 | Co-treatment |
| 5-ASA+Immunosuppressor | 1 | 1 |  |
| 5-ASA+Biologics | 2 | 0 |  |
| Steroids+Immunosuppressor | 3 | 4 |  |
| Steroids+Biologics | 1 | 0 |  |
| Immunosuppressor+Biologics | 1 | 5 |  |
| 5ASA+Immunosuppressor+Steroids | 1 | 1 |  |
| 5ASA+Immunosuppressor+Biologics | 0 | 1 |  |
| Steroids+Immunosuppressor+Biologics | 1 | 4 |  |
| No Treatments | 2 | 2 | No Treatment |

**Supplementary table 3.** Percentage of female (upper panel) and male (lower panel) study individuals from Kazan in each BMI category (as defined by the WHO) and study arm (control, UC, and CD), and overall (all).

| Female | Control | UC | CD | All |
| --- | --- | --- | --- | --- |
| Underweight | 1.5% | 2.9% | 10.3% | 14.7% |
| Normal weight | 16.2% | 16.2% | 20.6% | 52.9% |
| Pre-obesity | 7.4% | 7.4% | 1.5% | 16.2% |
| Obese class I/II/III | 5.9% | 10.3% | 0.0% | 16.2% |
| Male |  |  |  |  |
| Underweight | 0.0% | 0.0% | 5.8% | 5.8% |
| Normal weight | 21.2% | 27.3% | 50.0% | 55.8% |
| Pre-obesity | 11.5% | 9.6% | 7.7% | 28.8% |
| Obese class I/II/III | 3.8% | 1.9% | 3.8% | 9.6% |

**Supplementary table 4.** Distribution of positive/negative samples in the study groups. Anti-PR3 (cANCA).

|  | Negative | Positive |
| --- | --- | --- |
| Control | 42 | 0 |
| CD | 38 | 3 |
| UC | 36 | 7 |

**Supplementary table 5.** Distribution of positive/negative samples in the study groups. Human anti-*Saccharomyces cerevisiae* antibody **(A)** IgA and **(B)** IgG.

A

|  | Negative | Positive |
| --- | --- | --- |
| Control | 41 | 1 |
| CD | 19 | 22 |
| UC | 40 | 3 |

B

|  | Negative | Positive |
| --- | --- | --- |
| Control | 42 | 0 |
| CD | 36 | 5 |
| UC | 43 | 0 |

# **References:**

1. Benjamini Y, Hochberg Y. Controlling the false discovery rate: A practical and powerful approach to multiple testing. *Journal of the Royal statistical society: series B (Methodological)* 1995;**57**:289-300.

2. Meadow JF, Altrichter AE, Kembel SW*, et al.* Indoor airborne bacterial communities are influenced by ventilation, occupancy, and outdoor air source. *Indoor air* 2014;**24**:41-8.

3. Schloss PD, Westcott SL, Ryabin T*, et al.* Introducing mothur: Open-source, platform-independent, community-supported software for describing and comparing microbial communities. *Appl Environ Microbiol* 2009;**75**:7537-41.

4. Caporaso JG, Kuczynski J, Stombaugh J*, et al.* Qiime allows analysis of high-throughput community sequencing data. *Nature methods* 2010;**7**:335-6.

5. Edgar RC, Haas BJ, Clemente JC, Quince C, Knight R. Uchime improves sensitivity and speed of chimera detection. *Bioinformatics (Oxford, England)* 2011;**27**:2194-200.

6. DeSantis TZ, Hugenholtz P, Larsen N*, et al.* Greengenes, a chimera-checked 16s rrna gene database and workbench compatible with arb. *Appl Environ Microbiol* 2006;**72**:5069-72.

7. McIver LJ, Abu-Ali G, Franzosa EA*, et al.* Biobakery: A meta'omic analysis environment. *Bioinformatics (Oxford, England)* 2018;**34**:1235-7.

8. Franzosa EA, McIver LJ, Rahnavard G*, et al.* Species-level functional profiling of metagenomes and metatranscriptomes. *Nature methods* 2018;**15**:962-8.
