# Supplementary figures and images for: Inflammatory Bowel Disease–Associated Changes in the Gut: Focus on Kazan Patients
Source: Inflamm Bowel Dis. 2020 Aug 7;27(3):418–33. doi: 10.1093/ibd/izaa188 (PMC7885336; doi:10.1093/ibd/izaa188)

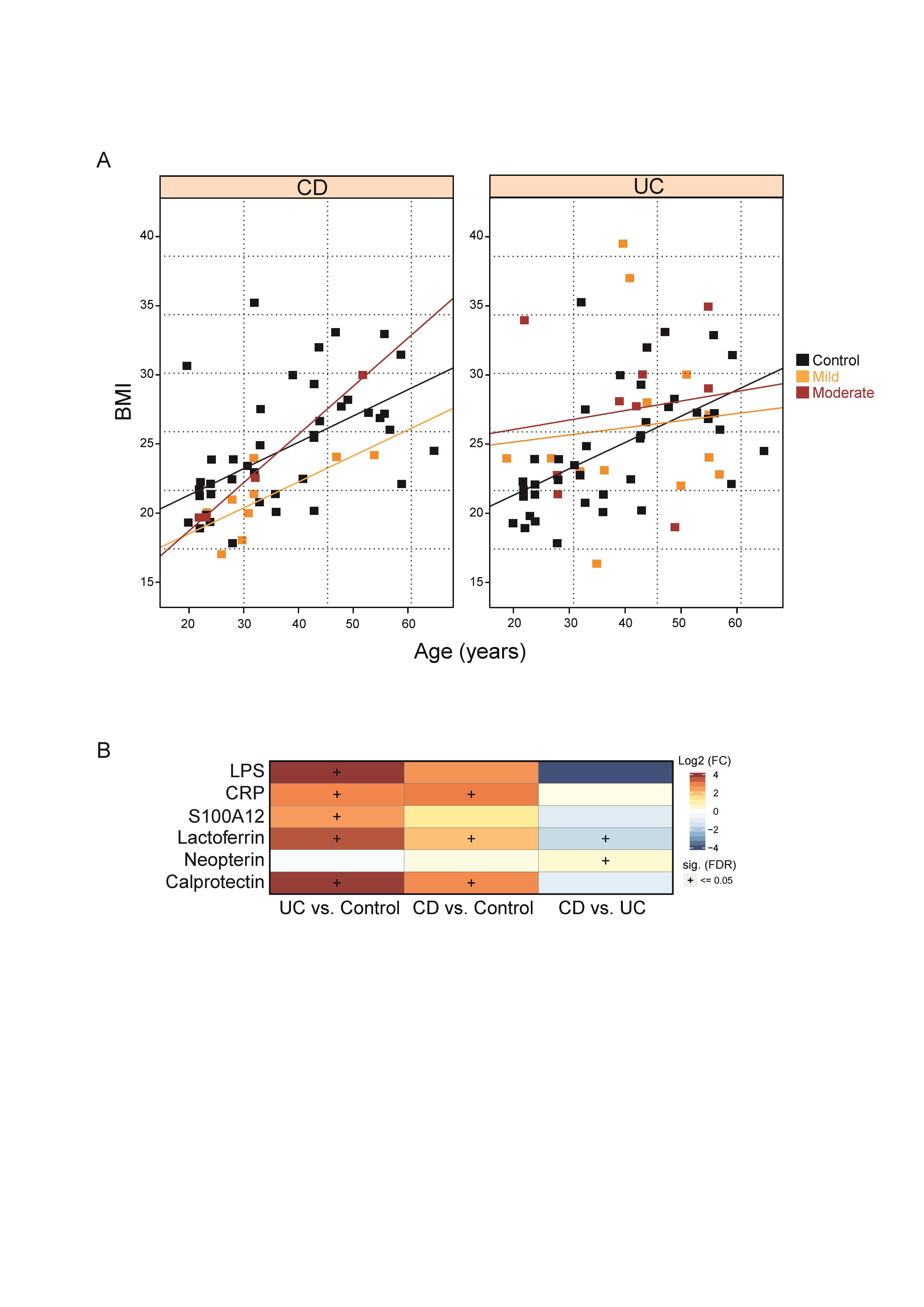

Supplement: izaa188_suppl_Supplementary_Figure_1 [file izaa188_suppl_supplementary_figure_1.jpeg]

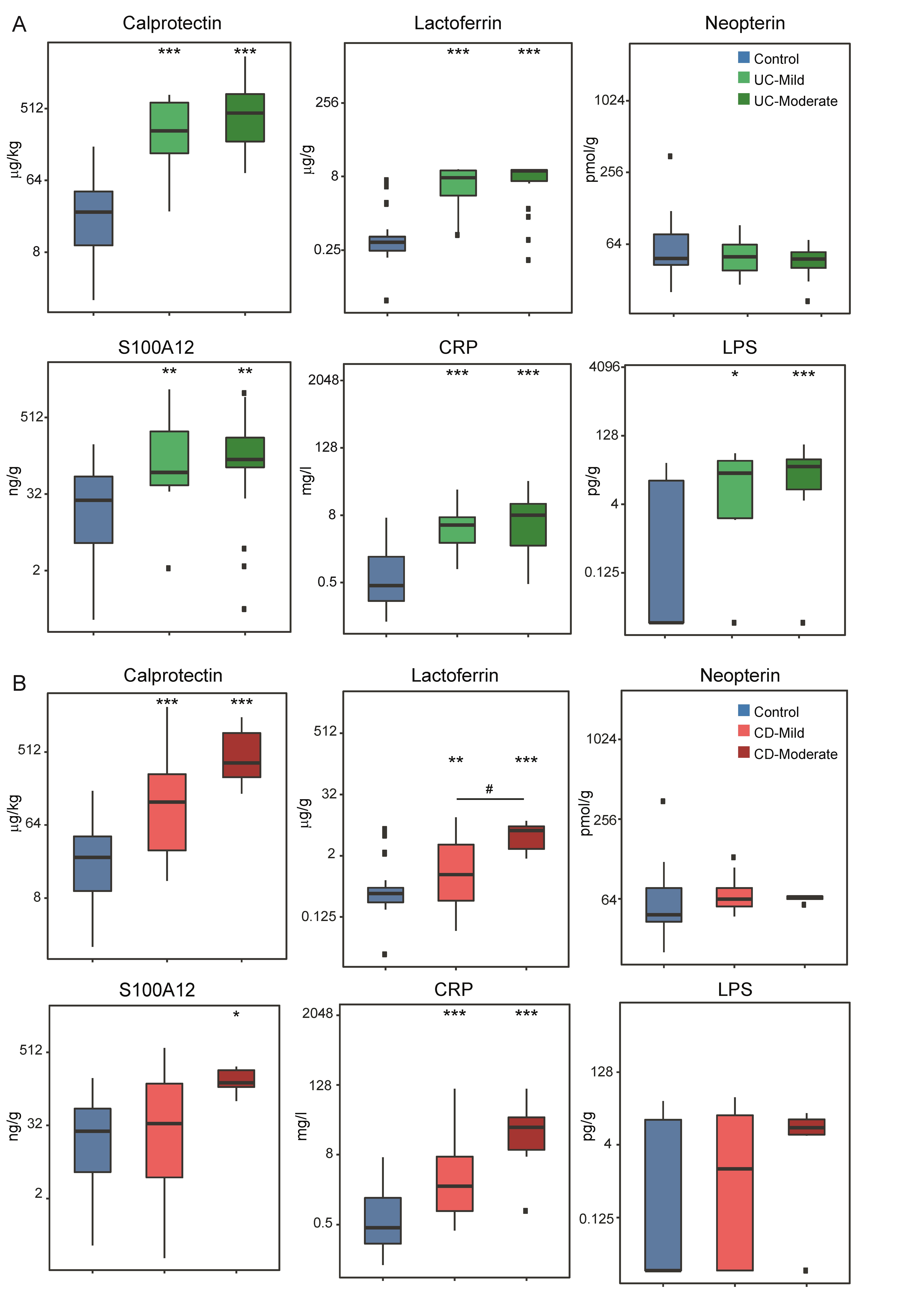

Supplement: izaa188_suppl_Supplementary_Figure_2 [file izaa188_suppl_supplementary_figure_2.jpeg]

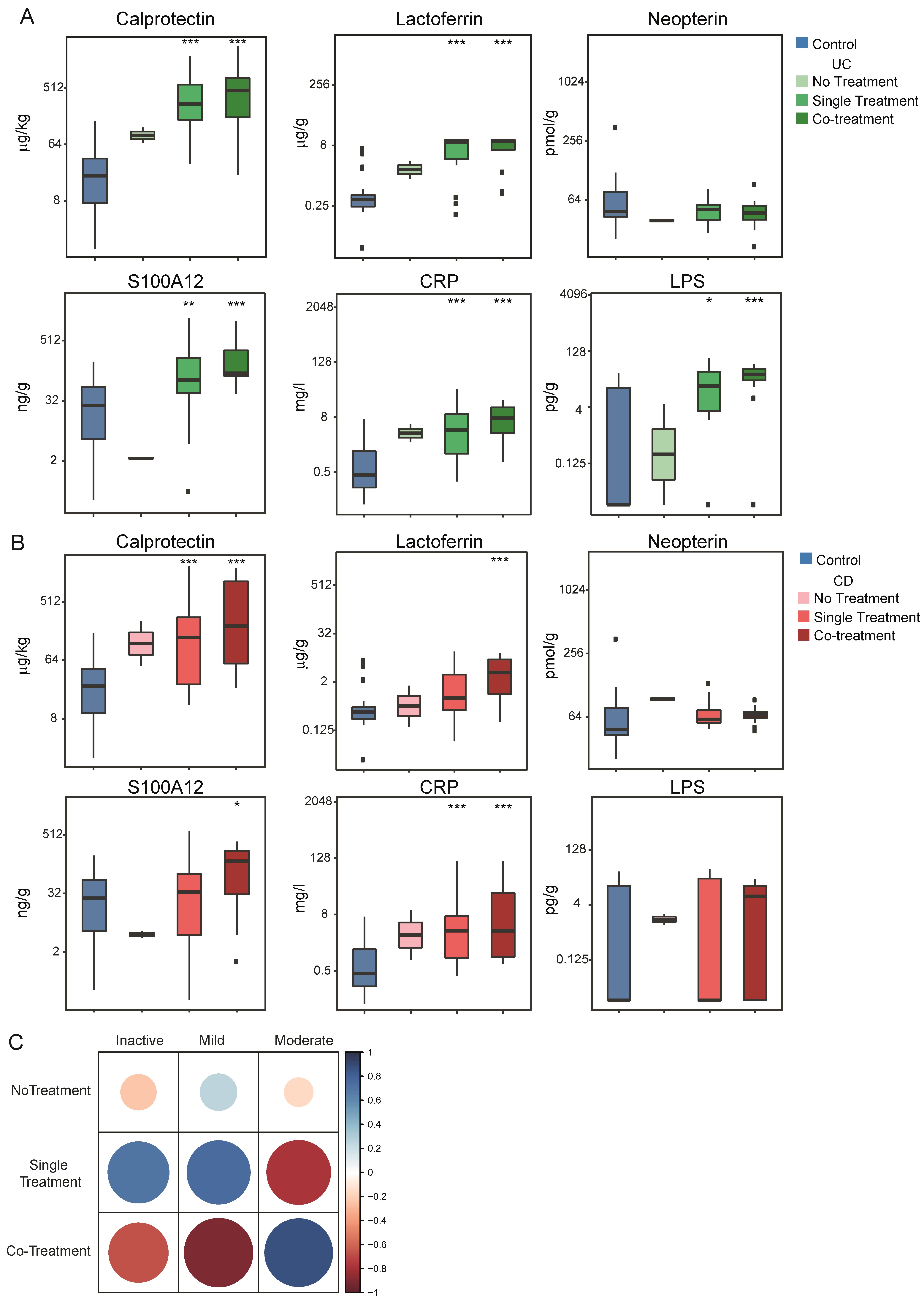

Supplement: izaa188_suppl_Supplementary_Figure_3 [file izaa188_suppl_supplementary_figure_3.jpeg]

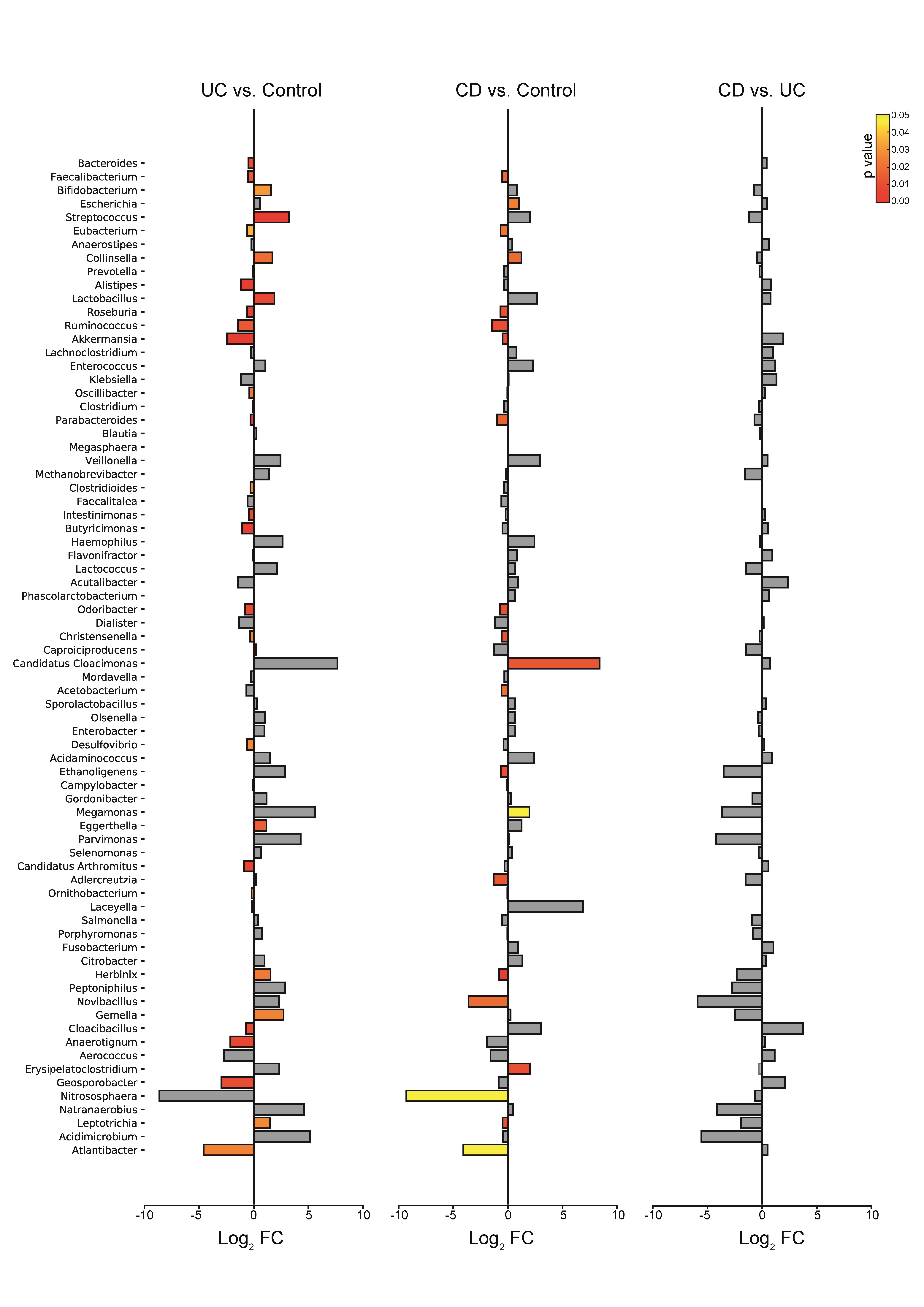

Supplement: izaa188_suppl_Supplementary_Figure_4 [file izaa188_suppl_supplementary_figure_4.jpeg]

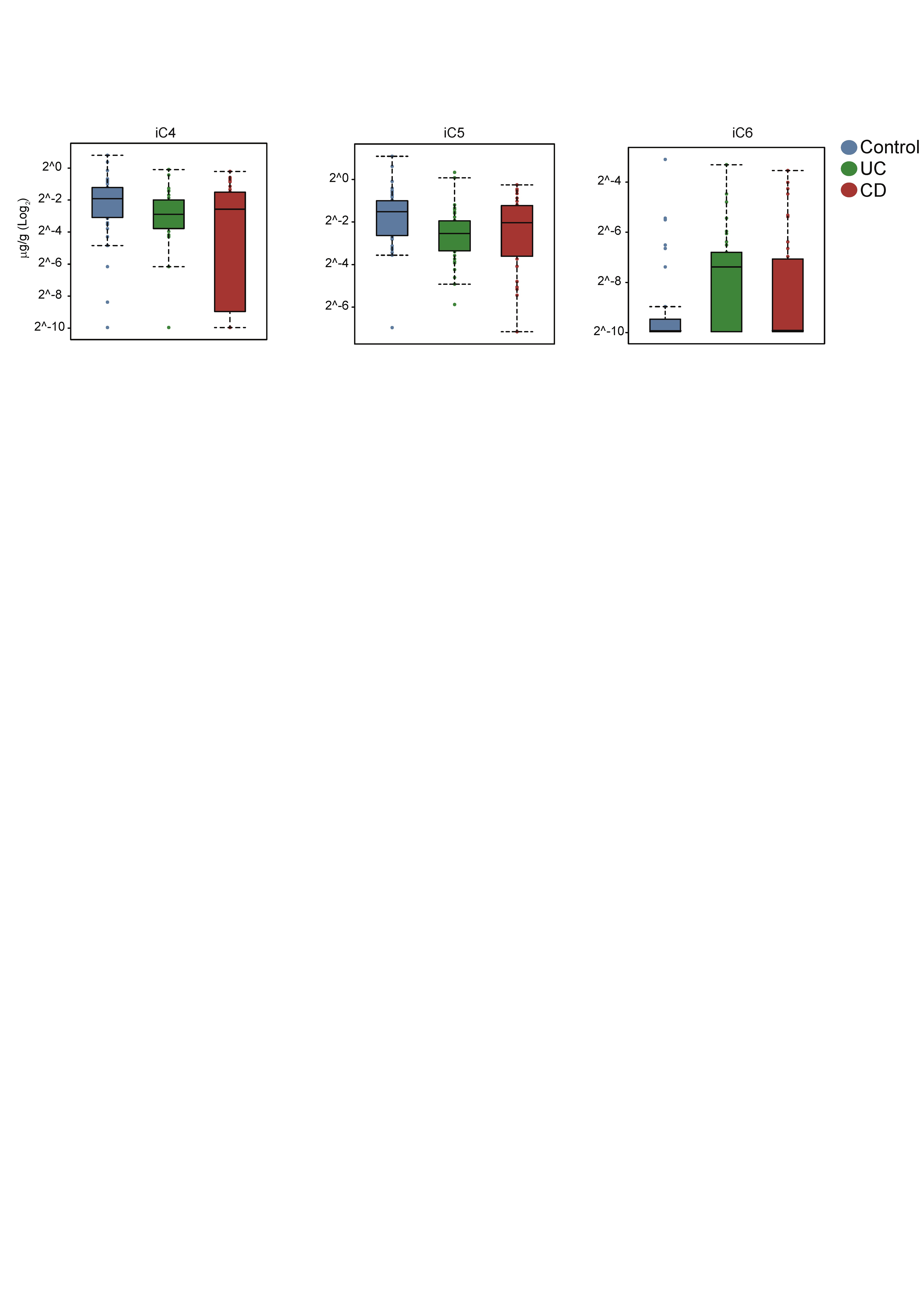

Supplement: izaa188_suppl_Supplementary_Figure_5 [file izaa188_suppl_supplementary_figure_5.jpeg]

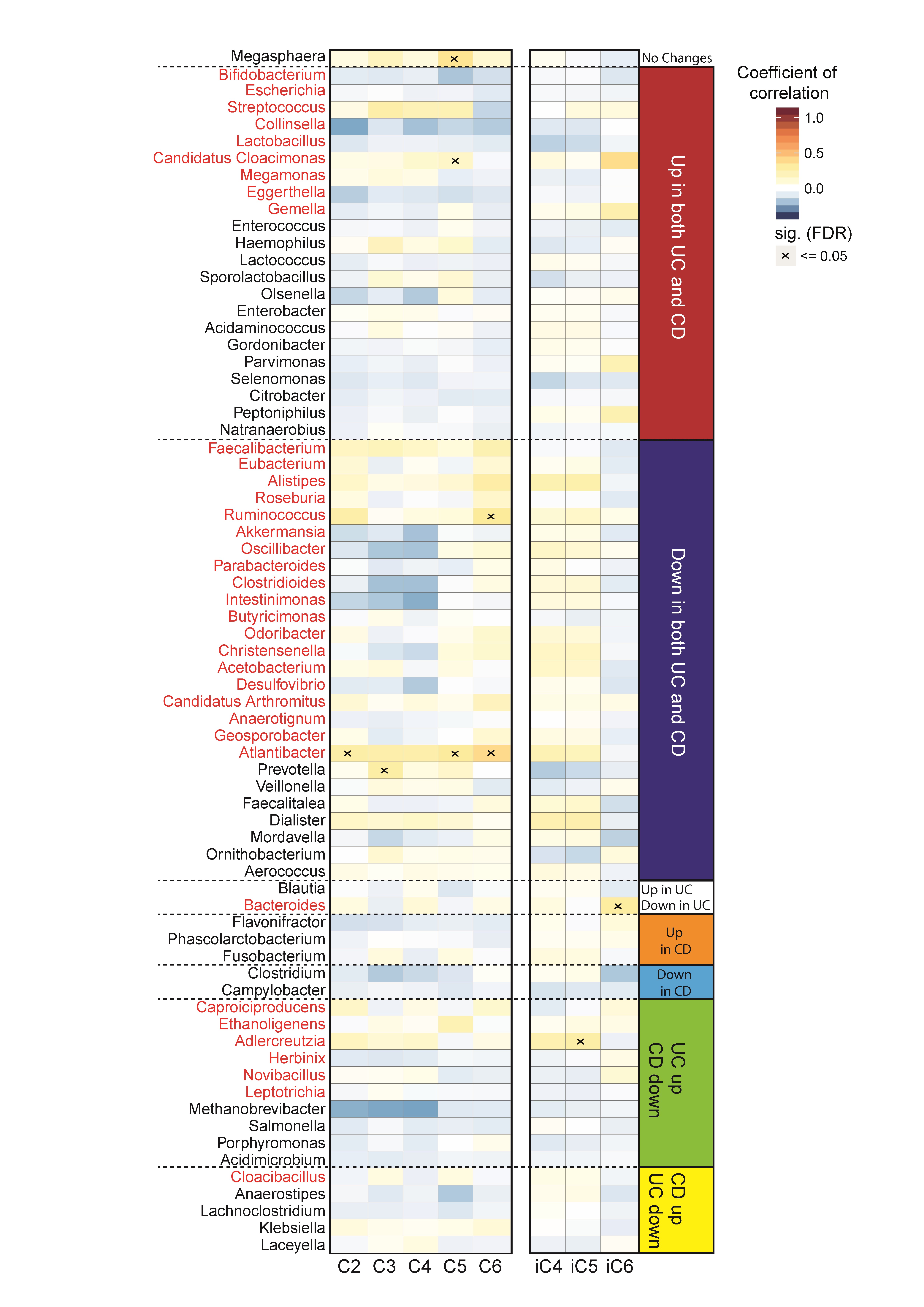

Supplement: izaa188_suppl_Supplementary_Figure_6 [file izaa188_suppl_supplementary_figure_6.jpeg]

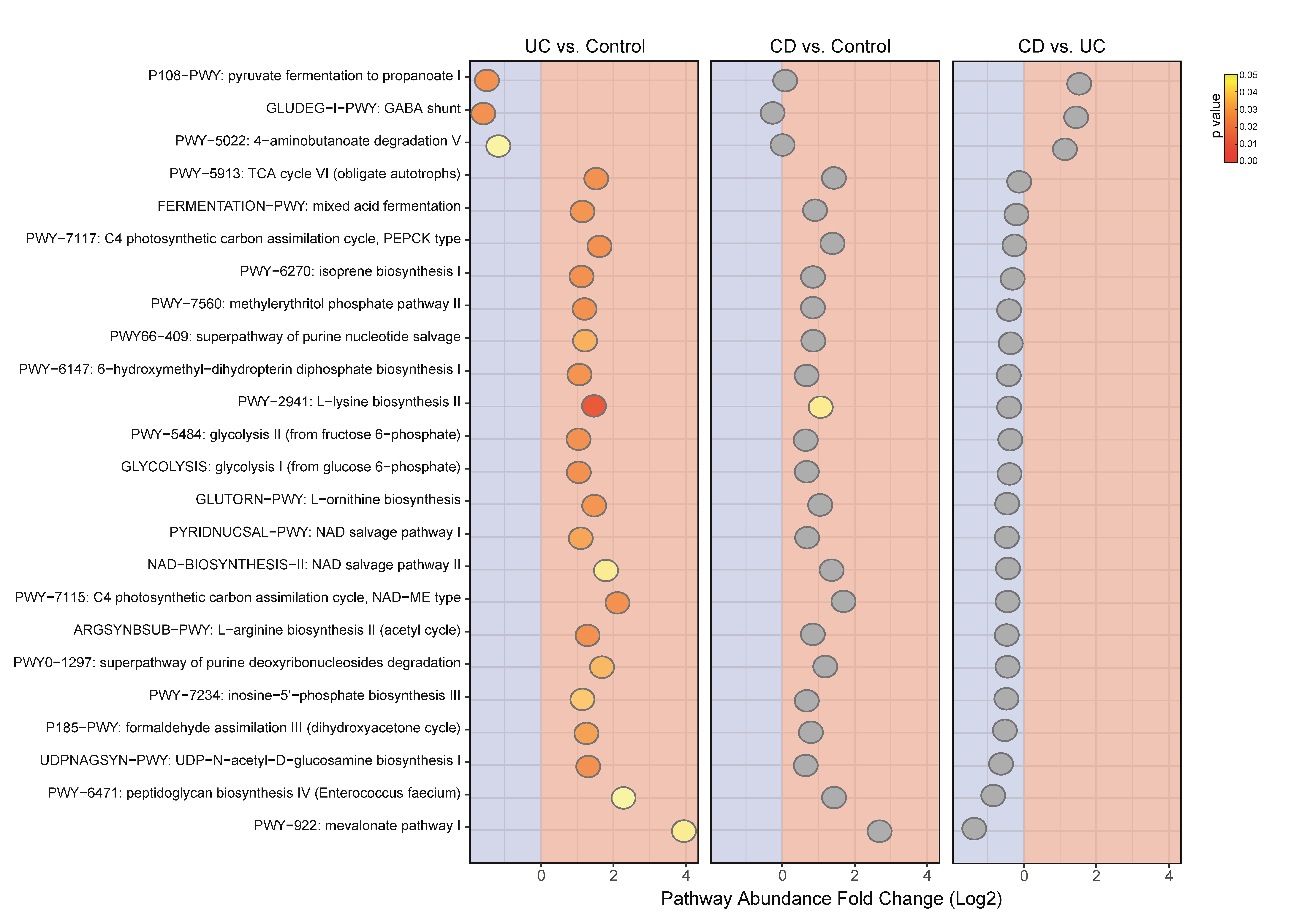

Supplement: izaa188_suppl_Supplementary_Figure_7 [file izaa188_suppl_supplementary_figure_7.jpeg]
